# A Comprehensive *in Silico* Analysis of Regulatory SNPs of Human CLEC7A Gene and Its Validation as Genotypic and Phenotypic Disease Marker in Recurrent Vulvovaginal Infections

**DOI:** 10.3389/fcimb.2018.00065

**Published:** 2018-03-20

**Authors:** Namarta Kalia, Manpreet Kaur, Sujata Sharma, Jatinder Singh

**Affiliations:** ^1^Department of Molecular Biology & Biochemistry, Guru Nanak Dev University, Amritsar, India; ^2^Department of Human Genetics, Guru Nanak Dev University, Amritsar, India; ^3^Department of Gynaecology & Obstetrics, Bebe Nanki Mother and Child Care Centre, Government Medical College, Amritsar, India

**Keywords:** bacterial vaginosis, serum Dectin-1(sDectin-1), mannose binding Lectin, mixed infections, PCR-RFLP, vulvovaginal candidiasis

## Abstract

Recurrent Vulvovaginal infections (RVVI) are the commonly reported microbiological syndrome affecting millions of women globally. Various molecules of innate immune system are instrumental in clearance of these microbial pathogens, thus suggested as one of the most important contributing factor in determining the disease outcome. Dendritic cell-associated C-type lectin-1 (Dectin-1) is an important molecule of innate immunity that is primarily known for its role in antifungal defenses. However, role of dectin-1 in recognition of other pathogens is also documented. The intracellular expression of dectin-1 was shown to be up-regulated by Mannose Binding Lectin (MBL)-mediated opsonophagocytosis of pathogens. Dectin-1 is encoded by *CLEC7A*, postulated to be a candidate gene in modulating risk of developing RVVI. In this study, we identified *CLEC7A* causal variants using *in silico* analysis. To assess their impact on susceptibility to RVVI, these causal variants along with serum dectin-1 levels (sDectin-1) were investigated using polymerase chain reaction-restriction fragment length polymorphism (PCR–RFLP) and Enzyme Linked Immnosorbent Assay (ELISA) respectively, under a case-control design. Furthermore, effect of these polymorphisms was also assessed on sMBL levels. *In silico* analysis revealed 9 putative functional conserved SNPs of *CLEC7A*. Association analysis revealed a significantly lower risk of developing RVVI and its types in carriers of *CLEC7A* rs3901533 G allele and its homozygous genotypes (*p* < 0.05). The heterozygous genotype was associated with significant protection against RVVI (*p* = 0.004). Haplotypes GGG and GTA showed significant protection against RVVI (*p* < 0.0001; *p* = 0.0003), Bacterial Vaginosis (*p* = 0.03; *p* = 0.002), Vulvovaginal Candidiasis (*p* = 0.03; *p* = 0.01) and Mixed Infections (*p* = 0.007; *p* = 0.04). Mean sDectin-1 levels were significantly high in RVVI and its types compared to controls (*p* < 0.05). Further, genotype-phenotype stratification showed significant differences within/between cases groups and controls. The *CLEC7A* rs3901533 polymorphism was also found to be associated with sMBL levels. The present study contributed novel insights into the role of dectin-1 in RVVI. *CLEC7A* rs3901533 polymorphism and high sDectin-1 levels along with low sMBL levels were found to be associated with RVVI susceptibility. Thus, screening of women with RVVI for these novel associations may lead to better diagnosis and treatment. Also genotyping method used in this study constitutes a simple and reliable assay, which can be confidently, used as a cheaper alternative for genotyping these variants in clinical settings. Finally, new restorative markers for other infectious diseases might be found by exploring nine functionally identified *CLEC7A* SNPs.

## Introduction

Vulvovaginal infections (VVI) are the commonly reported microbiological syndrome affecting millions of women globally in all strata of society. An abnormal vaginal discharge is a key trait of VVI and its estimated prevalence in India is 30% (Thulkar et al., 2010). Bacterial Vaginosis (BV), Vulvovaginal Candidiasis (VVC), and Trichomoniasis (TV) are the three main causes of VVI (Mulu et al., 2015). Mutual existence of these causes is termed as Mixed Infections (MI) that contribute >20% of women with VVI (French et al., 2004; Kalia et al., 2015). Besides this, cases of recurrent VVI (RVVI) have also emerged that is commonly stated as repeated experiences of vaginal infections in a definite period, this includes recurrent VVC (RVVC) and recurrent BV (RBV). RVVC refers to ≥4 repeated episodes of VVC in 12-months while RBV refers to the repeated episodes of BV within 3 months with recurrence rates as high as 30–50% (Powell and Nyirjesy, 2014).

**Figure 1:**
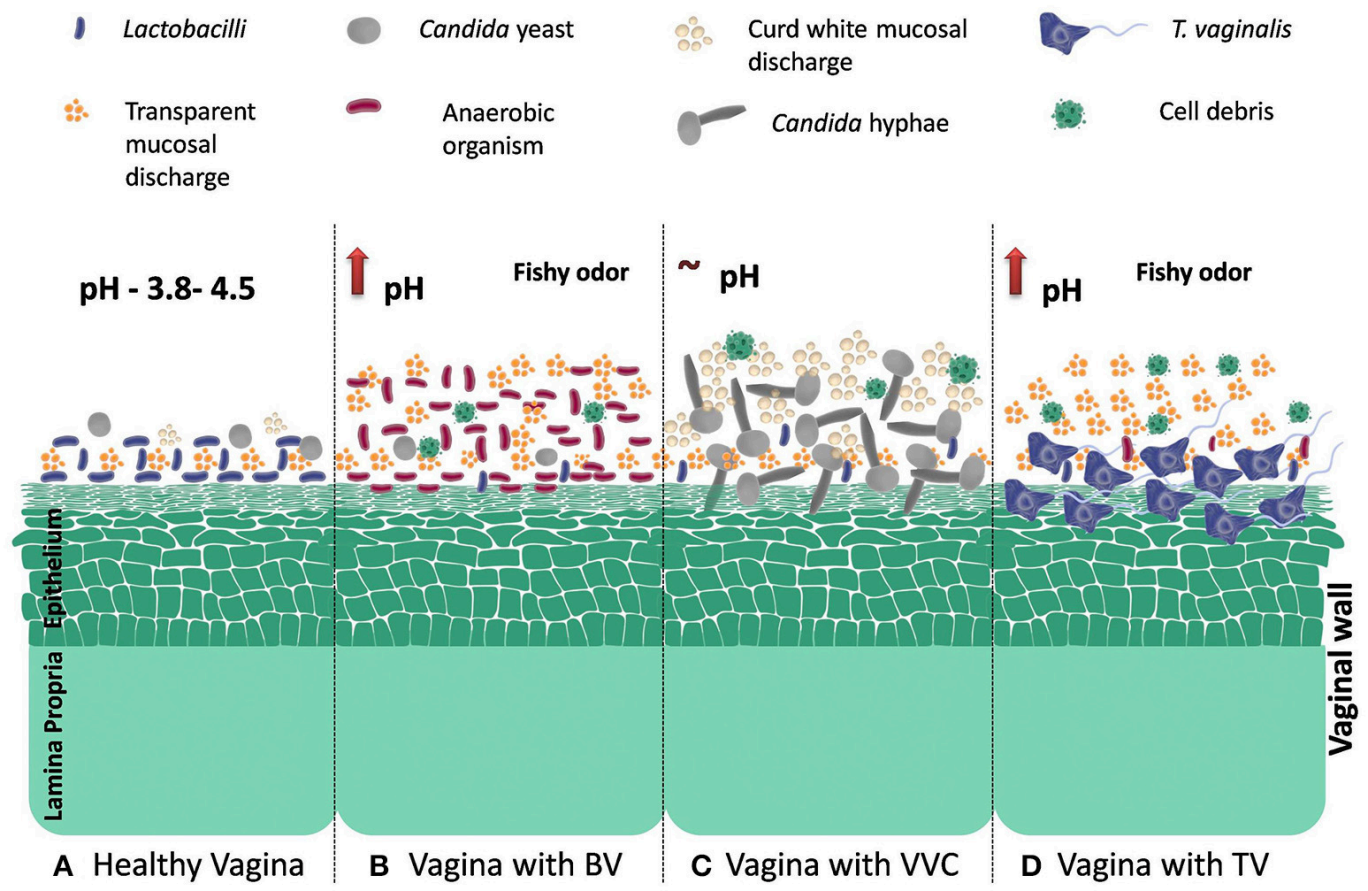
Model of vaginal microbiome disruption and induced conditions during RVVI **(A)**. Healthy vaginal microbiome: abundance of *Lactobacillus*- the Gram-positive rods, a low pH (3.5–4.5), small numbers of *Candida* species- a dimorphic yeast, small volume of white clear flocculent fluid and the absence of both facultative and obligatory anaerobic Gram-negative rods. Microbiome during RVVI **(B)** BV: is typified by decrease in hydrogen peroxide producing *Lactobacilli*, overgrowth of predominantly anaerobic organisms of the genera *Atopobium, Prevotella, Mobiluncus*, and *Sneathia* in the vagina, increase vaginal discharge with fishy odor and rise in pH. **(C)** VVC: decrease in hydrogen peroxide producing *Lactobacilli* and overgrowth of *C. albicans* that undergo morphogenetic change from yeast cell to a hyphal mycelial growing organism (dimorphic transition). These hyphae strongly adhere to, and then invade, the outermost layer of the vaginal epithelium. Detachment of the Hyphae from the epithelium, together with recruited inflammatory cells, debris from lysed cells and vaginal fluid make up the curd white vaginal discharge **(D)** TV: adhesion of trichomonads to the epithelial cells in the vaginal environment is a critical step in the pathogenesis of this parasite. Also increase vaginal discharge with fishy odor and rise in pH.

Literature regarding RVVI pathogenesis suggests that it is caused by a fall in hydrogen peroxide producing lactobacilli and overgrowth of microbes that are either normally present in human vaginal microbiome in lower quantity or sexually transmitted. Pictorial representation of various pathophysiological variations in vaginal flora during RVVI is depicted in Figure 1 (Sobel, 1988; Forsum et al., 2005; Hickey et al., 2012; Li J. et al., 2012). In addition, use of contraceptives, excessive antibiotics, smoking, sexual activity, immunosuppression, and black race are the other known predisposing factors that leads to RVVI (Bleicher and Stockdale, 2015; Sobel, 2016). Untreated RVVI can lead to complications like infertility, pre-term birth, miscarriage, vulvovaginal inflammation, and other infectious diseases (Hay et al., 1994; McClelland et al., 2007; Atashili et al., 2008; Toth et al., 2015). Though diverse microbial strains have been defined in literature as a causative agent for RVVI, till date no major determinants have been recognized that could explain susceptibility to this disease condition (van de Wijgert et al., 2014). So, it was hypothesized that RVVI susceptibility may be determined by individual's own genetic factors.

These genetic factors include pattern recognition receptors (PRRs) that identify preserved biomolecular structures on surface of pathogens also known as pathogen associated molecular patterns (PAMPs). This interaction further leads to the generation of specific immune responses against pathogens (Kumar et al., 2011; Santoni et al., 2015). The toll-like receptor (TLR) family of PRRs contributes crucially in pathogen recognition and immune responses generation. However, the attention has now been restored to non-TLR PRRs, particularly C-type lectin receptors (CLRs) which are Ca^2+^-dependent carbohydrate binding proteins. Of these, the one typical member is Dendritic cell-associated C-type lectin-1 (Dectin-1) (Brown, 2006).

Human dectin-1 is expressed as a monomer both on surface as well as in cytoplasm of myeloid cells and lymphocytes (Brown, 2006). It is a 28 kDa glycosylated type II transmembrane receptor encoded by *CLEC7A* mapped to 12p13.2 (Ariizumi et al., 2000). Differential splicing of precursor mRNA of dectin-1 leads to the formation of two chief isoforms A and B, both having surface expression but different functionalities (Willment et al., 2001, 2005; Heinsbroek et al., 2006; Del Pilar Jimenez et al., 2008). Isoform A has a surface C-type lectin-like domain (CTLD) that is attached to an intracellular immune receptor tyrosine-based activation motif (ITAM) via stalk and region across the membrane. However, isoform B is characterized by absence of stalk. Alternative splicing also generates six minor isoforms of dectin-1, one of which is isoform E. This isoform do not have stalk and transmembrane region, thus have cytoplasmic expression and interaction with other cytosolic proteins (Xie et al., 2006). However, the function of other minor isoforms remains poorly stated.

Dectin-1 recognize 1,3-β-glucans on the surface of pathogens including fungi and some bacteria. This receptor ligand interaction leads to intracellular signaling through Raf-1 and Syk-CARD9 pathway, that further causes pro-inflammatory signaling proteins expression and cytotoxic T-cell responses (LeibundGut-Landmann et al., 2007; Gerosa et al., 2008; Heinsbroek et al., 2008; Gringhuis et al., 2009). Dectin-1 is primarily known for its role in antifungal defenses. However, role of dectin-1 in recognition of other pathogens including *Mycobacterium tuberculosis, Salmonella typhimurium, Leishmania infantum, and Haemophilus influenzae* is also documented (Drummond and Brown, 2013; Lefèvre et al., 2013; Heyl et al., 2014). However, these pathogens do not possess β-glucans, suggesting the possibility of other ligands of Dectin-1 that are still not revealed.

Interestingly, a study has highlighted the specific role of *CLEC7A* rs16910526 (Y238X) polymorphism in antifungal defenses in four women from Netherlands, affected either by onychomycosis or RVVC (Ferwerda et al., 2009). However, no correlation of this polymorphism was found in susceptibility to RVVC in three different populations i.e., Turkish, western-european, and Iranian (Rosentul et al., 2014; Usluogullari et al., 2014; Zahedi et al., 2016). Irrespective of the prime role of dectin-1 in antifungal activity, its aforesaid role in recognition and generation of immune responses against bacterial and other pathogens cannot be ignored. This led us to evaluate the role of *CLEC7A* polymorphisms in RVVI and its types that has not been investigated earlier. Also, functional implications of only few SNPs of *CLEC7A* are known out of 1179 SNPs in dbSNP, as of October, 2016 while majority of SNPs are still not established (Sherry et al., 2001; Bhagwat, 2010). Therefore, to predict the putative functional consequences, all the SNPs of *CLEC7A* were analyzed using different bioinformatic tools (Bhatti et al., 2006; Johnson, 2009; Li and Wei, 2015). Also, studies have suggested the release of cytosolic proteins from the cells by non-classical secretary pathways (Cooper and Barondes, 1990; Kuchler and Thorner, 1990; Muesch et al., 1990; Muthukrishnan et al., 1991). Furthermore, it was proposed that Dectin-1/Syk signaling pathway leads to protein secretion by non-classical secretary means in human macrophages (Öhman et al., 2014). This directed us to determine the unconventional release of cytoplasmic form of dectin-1 in serum (sDectin-1) of RVVI cases and controls.

Moreover, Dectin-1 has been shown to collaborate with other PRRs including TLRs and CLRs through Syk pathway to induce optimal immune responses (Brown, 2006). Human Mannose-Binding Lectin (MBL) is a CLR that provides defense against a variety of pathogens (Eisen and Minchinton, 2003; Brouwer et al., 2008; Kasperkiewicz et al., 2015; Kalia et al., 2017). Our previous findings have indicated the association of low sMBL levels with susceptibility to RVVI (Kalia et al., 2017). Both dectin-1 and MBL are the important components of innate immunity. However, how these two PRRs interact with each other, when co-activated, has not been reported till date. The only study that so far collaborate the two PRRs has shown that intracellular expression of dectin-1 increases with MBL-arbitrated opsonophagocytosis of pathogens (Li D. et al., 2012). This may subsequently lead to increased serum concentration of dectin-1 by unconventional release of its increased intracellular protein (Öhman et al., 2014). To find the relationship between sDectin-1 and sMBL, if any, the correlation analysis was carried out. In addition it has been reported that variations in dectin-1 gene modulates its protein expression, ligand binding ability and thus generate non optimal immune responses through defective collaborations (Ferwerda et al., 2009). Using this rational, the association of *CLEC7A* polymorphisms was also explored with serum MBL (sMBL) levels.

Thus, the current investigation involves sorting of underlying variants from a pool of *CLEC7A* SNPs using *in silico* analysis. These functionally identified variants were further evaluated to assess their impact on susceptibility to RVVI and its types using a conventional approach. In addition, we have also investigated the serum levels of dectin-1 (sDectin-1) in cases and controls. Furthermore, sDectin-1 and sMBL levels were also correlated with *CLEC7A* polymorphisms. All these findings are reported for the first time in the present study.

## Materials and methods

### Ethics issues

The present study was commenced after getting approval from the Institutional Ethics Committee (Approval no. 06/HG dated 02/01/2015) of Guru Nanak Dev University, Amritsar (Punjab), India, in accordance with Indian Council of Medical Research guidelines (ICMR, 2006) modified from World Medical Association (2004). Voluntary consent in written was attained from all the subjects. To ensure that subjects involved in the study had necessary information to make an informed choice the information provided to them include comprehensive depiction of the present study purpose, confirmation of secrecy of their provided information, vaginal, and blood samples collection procedures and possible risks and benefits of the study. The necessary information was recorded in a pre-designed Proforma. In addition, the bio-medical waste was segregated and managed on the basis of color coding as mentioned in Bio-Medical Waste (Management & Handling) Rules, 1998 (amended in 2000).

### Subjects and sample processing

The study included 258 RVVI cases (Age, mean ± S.E., 29.33 ± 0.51 years) from the Department of Gynaecology and Obstetrics, Bebe Nanki Mother and Child Care Centre, Government Medical College, Amritsar (Punjab). These recommended cases by gynecologist were clinically diagnosed with RVVI with minimum four documented recurrent experiences in a year. These cases complained of having frequent symptoms like vaginal fishy smell, burning, discharge, itching, pelvic pain, pruitis, and soreness. Participants with immunodeficiencies, using immunosuppressive medications, under chemotherapy or having HIV infections were excluded from the study. For further confirmation, 200 RVVI cases were processed on the basis of standard tests given in European (IUSTI/WHO) guidelines on vaginal discharge management (Sherrard et al., 2011). The particulars of vaginal discharge sample collection as well as processing, diagnosis of various types of RVVI along with clinical characteristics of RVVI cases have been reported previously (Kalia et al., 2015, 2017). This categorized 200 RVVI cases into three classes including BV (*N* = 97), VVC (*N* = 62) and Mixed Infections (MI; N = 41). However vaginal samples from the rest of 58 cases could not be processed as some participants were reluctant to give vaginal samples and others were menstruating. Thus, these participants were not sub categorized into RVVI categories. Age matched healthy women (mean ± S.E., 29.33 ± 0.57 years, N = 203) forms the control group. These women do not have any recurrent history of vaginal infection complaints. Peripheral blood samples (5 ml) were collected from each study participant. 2.5 ml of this blood was transferred to EDTA (0.5 M) coated vial for genomic DNA isolation and the other half was transferred to plain vial without anticoagulant for serum separation. Genomic DNA isolation was performed using inorganic method by Miller et al. (1988) from peripheral blood mononuclear cells (PBMCs). Serum separation from blood was performed by incubating the samples for 1 h at 37°C followed by 10 min centrifugation (Spinwin, Tarson, India) at 300 × g and serum was collected as supernatant. These isolated samples both DNA and serum samples were stored at −80°C till further analysis.

### *In silico* analysis of human *CLEC7A* polymorphisms

The data mining for SNPs in Human *CLEC7A* was carried out using dbSNP database (http://www.ncbi.nlm.nih.gov/SNP/). These SNPs were put through different bioinformatics tools analysis. The details of computational tools used, along with scheme and criteria adopted to select the functional SNPs were reported previously (Kalia et al., 2016). In brief, to verify the consistency of a particular SNP in dbSNP database, all the SNPs were primarily looked for validation status. Secondly, Minor Allele Frequency (MAF) was used to further prioritize the SNPs having MAF ≥ 0.10. Further, Ensembl Genome browser release 48 (http://www.ensembl.org/) was used to recognize SNPs located in evolutionary conserved regions (cSNPs). For this, relative analysis of human *CLEC7A* was performed with seventeen eutherian mammals including *Bos Taurus, Canis lupus familiaris, Callithrix jacchus, Chlorocebus sabaeus, Equus caballus, Felis catus, Gorilla gorilla gorilla, Macaca mulatta, Mus musculus, Ovis aries, Oryctolagus cuniculus, Pan troglodytes, Pongoabelii, Papio Anubis, Rattus norvegicus*, and *Sus scrofa*. As, no non-synonymous SNPs (nsSNPs) of *CLEC7A* were found to have MAF ≥ 0.10, only tools for functional analysis of non-coding SNPs were used. These include SNPinfo (FuncPred; https://snpinfo.niehs.nih.gov/snpinfo/snpfunc.html), RegulomeDB (http://regulomedb.org/) and PolymiRTS (v3.0) (http://compbio.uthsc.edu/miRSNP/). SNPinfo comprises of different sets of data processing elements for the selection of functional SNPs. FuncPred, the main tool of SNPinfo is composite of different singleton tools including SNP3D, rescue ESE, MATCH, miRanda, Polyphen, TRANSFAC12.1, miRBase, FAS–ESS, and ESEfinder (Xu and Taylor, 2009). RegulomeDB divides functional SNPs into six different classes ranging from C1 to C6. Class 1 SNPs mostly include expression and binding signals, with six subclasses from 1a to 1f that further categorize SNPs by falling confidence. Class 2 SNPs are interpreted as “likely to affect binding.” The other three classes (C4–C6) correspond to weak or minimal binding evidence for the functional SNPs (Boyle et al., 2012). PolymiRTS (v3.0) is used to recognize SNPs locating in miRNAs seed region and its complement binding sites (Bhattacharya et al., 2014).

### Polymerase chain reaction-restriction fragment length polymorphism (PCR–RFLP) for the validation of *CLEC7A* SNPs by genotyping

The present study was able to standardize PCR-RFLP, an easy low cost method, for three *CLEC7A* SNPs i.e., rs11053593, rs11053597, and rs3901533 to analyze their association with susceptibility to RVVI.

#### Design of PCR primers for PCR-RFLP

Primers for PCR amplification of rs11053593, rs11053597, and rs3901533 SNPs were designed using the online software Primer-BLAST (https://www.ncbi.nlm.nih.gov/tools/primer-blast/) and Primer3 (http://bioinfo.ut.ee/primer3-0.4.0/). The flanking sequence of the given SNP, based on the size of PCR product required, was obtained from the *CLEC7A* gene sequence (NCBI Reference Sequence: NC_000012.12). The best primer pair was selected based on the optimal GC content of 40–60% and the difference of GC content between forward and reverse primers <10%. The oligos were custom-synthesized by Bioserve Biotechnologies (Hyderabad, India). PCR was carried out using target DNA (3 ng/ul), deoxynucleotide triphosphate (0.05 mM each), appropriate forward and reverse primers (0.15 pmol each), Taq DNA polymerase (0.3 U) and polymerase buffer with 1.5 μM MgCl_2_ (1X) in 20 μl reaction volumes (GeNei, Bangalore). All PCRs were carried out in a thermocycler with pre-set conditions (Applied Biosystems, Life Technologies, USA). Primer sequences along with the PCR conditions used for each reaction are given in Table 1A. Ethidium bromide (SRL, India) stained agarose gel (1.5% w/v; Himedia, India) was prepared and amplified products were loaded in it. These products were visualized using gel documentation system (Alpha imagner, USA) after electrophoresis at 100 V.

**Table 1:** PCR-RFLP protocol for the detection of *CLEC7A* polymorphisms.

**(A) PCR primers and conditions**.					
**SNP**	**Primers**	**Sequence (5′ → 3′)**		**Conditions for both reactions**	**PCR product**
rs11053593^*^, rs11053597^*^	Forward	ACATGGCAAAACCCAGTCTC		94°C for 5 min, followed by 34 cycles (94°C for 30 s, 63°C for 30 s, and 72°C for 30 s) and a final elongation at 72°C for 5 min	551 bp
	Reverse	ACCCAAGAAAACCCATCTCC			
rs3901533^#^	Forward	CTGGGAAAGGCAAAGAGGGTT			614 bp
	Reverse	TTAGCCATTGTCTTCTCCTCCAA			
**(B) Restriction digestion conditions and expected fragments size**.					
**Polymorphic sites**	**Enzyme**	**RFLP conditions**	**Wild-type homozygotes (bp)**	**Variant homozygotes (bp)**	**Heterozygotes (bp)**
G>A (rs11053593)	*HindIII-HF*	4 h at 37°C	354+197	551	354+197+551
G>T (rs11053597)	*HaeIII*		325+226	551	325+226+551
T>G (rs3901533)	*ApoI-HF*		371+119+124	371+243	371+243+119+124

“*” *Denote SNPs reported at forward strand*,

“#” *denote SNPs reported at Reverse strand in NCBI dbSNP database*.

#### Choice of restriction enzymes for PCR-RFLP

**Figure 2:**
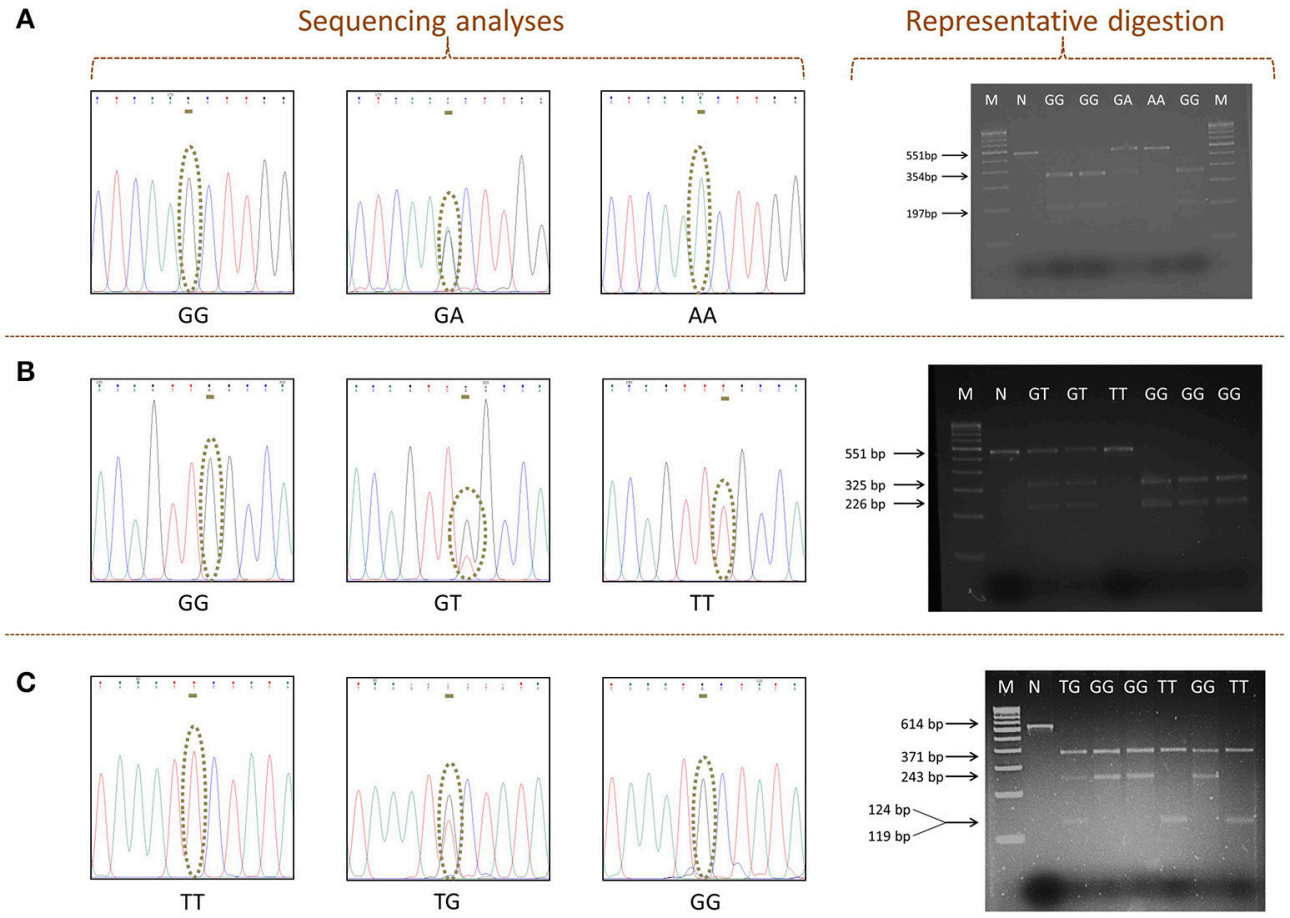
The detected polymorphisms of *CLEC7A*. Sequencing analyses indicating the presence of **(A)** rs11053593, **(B)** rs11053597, and **(C)** rs3901533. Representative digestion showing polymorphism. M: 100 bp DNA ladder (GeNei, Banglore). N: negative control (PCR product with no restriction enzymes).

The online tool NEBcutter v 2.0 (http://www.neb.uk.com/tools/index.aspx?req=nebcutter) was employed for the selection of restriction enzymes, which are able to distinguish between the wild and variant alleles related to SNPs rs11053593, rs11053597, and rs3901533 (Vincze et al., 2003). The simulation of DNA cutting by the proposed enzymes on gel and the analysis of the foreseen restriction profiles were also performed. Restriction endonuclease analysis of 20 μl of amplified PCR product was performed, using 1 unit of restriction enzyme and 1X NEBuffer (cut smart). Restriction endonucleases (New England BioLabs, Beverly, USA) chosen for each SNP along with the conditions considered are given in Table 1B. Restriction digestion was detected by 3% (w/v) agarose gel electrophoresis. Specific banding pattern of restricted PCR products obtained on gel was used to determine genotypes (Figure 2). Verification of representative genotypes was done by Sanger sequencing (Figure 2). These sequences were analyzed using the tool chromas 2.6.4 (http://technelysium.com.au/wp/chromas/).

### Serum concentrations of dectin-1 and MBL

Commercially available enzyme linked immnosorbent assay (ELISA) kits was used to quantify dectin-1 levels in serum according to manufacturer's instructions. This kit is capable of recognizing all isoforms of dectin-1 (Ray Biotech, USA). Detection limits were 0.082–20 ng/ml. The serum samples were pre-diluted 2 fold before use. Serum MBL (sMBL) quantification was also performed by commercially available ELISA kits as reported previously (Kalia et al., 2017).

### Statistics

The sample size, required for the present study with a power of 99% was determined by CaTS power calculator (http://csg.sph.umich.edu/abecasis/CaTS/) by taking conventions of odds ratio of 1.5, 5% significance level (*p* = 0.05) and 30% national prevalence of vaginal discharge. Allelic and genotypic frequencies of *CLEC7A* SNPs were calculated manually. Odds ratios (ORs) statistics at 95% confidence intervals was used to compare allelic and genotypic distribution of cases and controls. This statistical analysis was performed using MedCalc software v 9.3.9.0 (https://www.medcalc.org/). Inheritance models and hardy-Weinberg equilibrium were assessed by SNPStats (https://www.snpstats.net/snpstats/start.htm), a tool based on binary logistic regression analysis (Solé et al., 2006). The choice of best inheritance model for each SNP was made by selecting model with lowest score of Akaike's Information Criterion and Bayesian Information Criterion given by SNPStats. Haploview v 4.2 was used to perform linkage disequilibrium analysis (Barrett et al., 2004). PHASE software v 2.1.1 was used to construct and estimate haplotype frequencies (Stephens et al., 2001). *p*-values ≤ 0.05 were considered significant. The allele and corresponding homozygous genotype with highest frequencies in the total subjects were taken as reference (OR = 1). One way analysis of variance (ANOVA) followed by *post hoc* Tukey's test was performed to compare serum levels (mean ± S.E.M) within group. Student's *t*-test was used to compare serum levels of cases with respective controls. The correlation between serum biomarkers was calculated by Pearson's correlation coefficient analysis. SPSS statistical package v 16.0 was used for these statistical analyses.

## Results

### *In silico* analysis and SNPs selection

**Figure 3:**
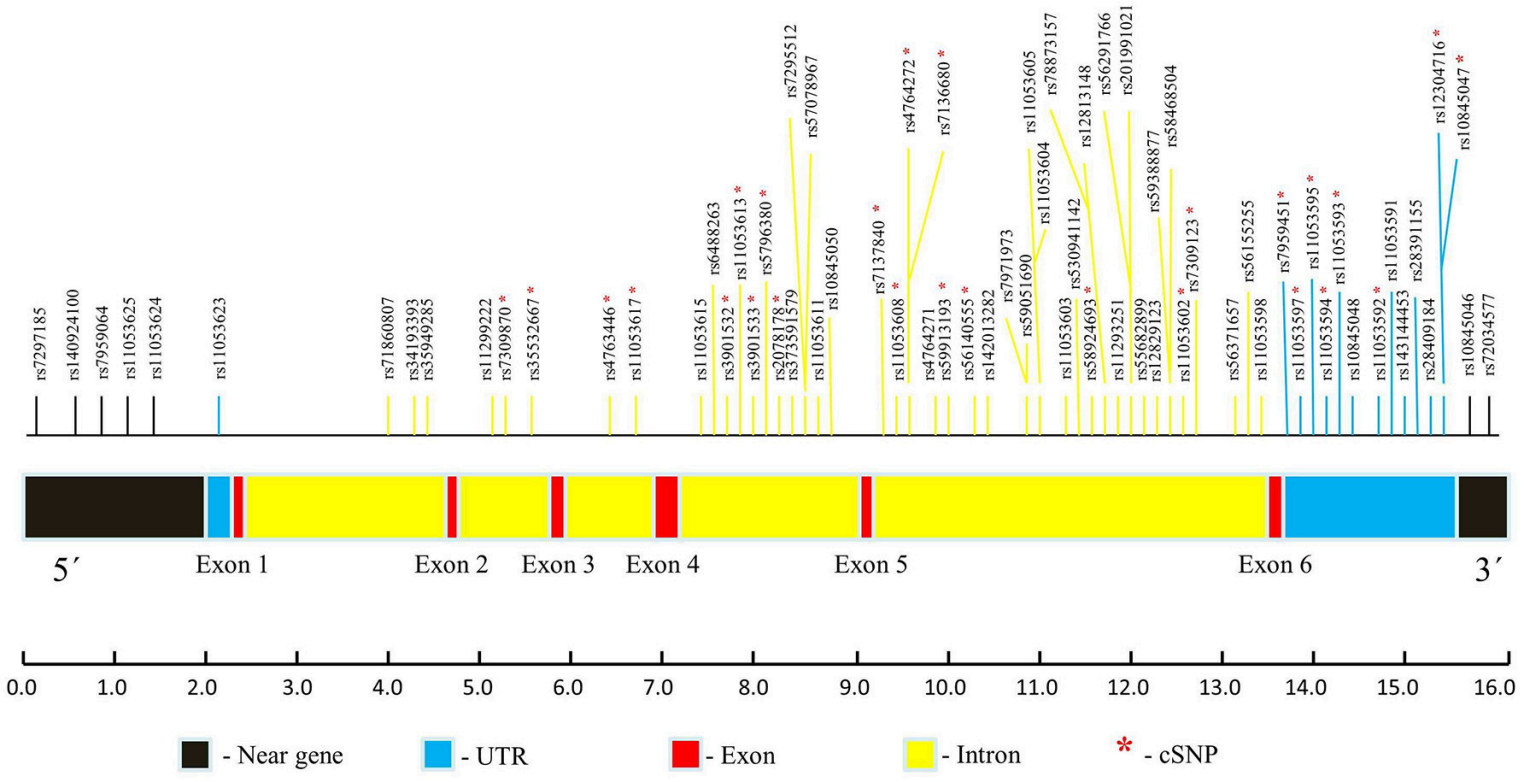
*CLEC7A* map pointing the locations of 70 validated SNPs (MAF ≥ 0.10) based on dbSNP database. The whole *CLEC7A* gene structure contains 6 exons and spans 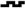 16.0 kb. Estimate distances of various *CLEC7A* regions are designated in bp underneath. ^*^indicate conserved SNPs (cSNPs).

The dbSNP is most considerable amongst all the available databases, though includes both validated as well as non-validated SNPs (Sherry et al., 2001; Bhagwat, 2010). Total 1,179 SNPs of *CLEC7A* are present in dbSNP in gene region view. Out of these, only 70 validated SNPs were found to have MAF ≥ 0.10. These SNPs comprised of only non-coding variants including two in 3′ near gene, thirteen in 3′ UTR region, forty nine in intronic region, one in 5′ UTR and five in 5′ near gene. Polymorphisms which are not validated or have MAF < 0.10 were omitted from further analysis. As no non-synonymous SNPs (nsSNPs) were found to have MAF even ≥0.05, so SNPs in clinical source view were also checked for validation status and MAF. However, the results remain same. Even most commonly studied nsSNP of *CLEC7A* i.e., Y238X (rs16910526) has MAF of 0.040, which was not fulfilling the criteria and hence omitted. Thus, the present analysis involves only 70 non-coding SNPs of *CLEC7A*. A *CLEC7A* map marking these SNPs positions is demonstrated in Figure 3.

In sequence alignment, all the species were found to show alignment with human *CLEC7A* except *C. jacchus, C. lupus familiaris, F.catus, O. cuniculus R. norvegicus*, and *M. musculus*. Only 26 conserved SNPs (cSNPs) were found, out of 70 non-coding SNPs of *CLEC7A* (Figure 3). These 26 cSNPs of *CLEC7A* falling in 3′ UTR and intron region were carried forward for further analysis in the present study. The results obtained after submitting 26 cSNPs to FuncPred are shown in Table S1. Total nine SNPs including eight in 3′UTR region and one in intronic region were predicted to be affecting miRNA binding sites. In addition, one 3′UTR SNP rs12304716 was found to be affecting splicing also. RegulomeDB was further used to analyse these 26 cSNPs, which provided an annotation scores for 19 SNPs ranging from 4 to 6, representing weak or minimal binding evidence for the functional SNPs (Table S2). The remaining seven SNPs get no data annotation (not shown in Table S2). The 26 cSNPs submitted to PolymiRTS predicted four SNPs of 3′UTR region altering miRNA target sites, thus suggesting their likelihood in resulting truncated proteins (Table S3). Furthermore the findings of FuncPred were replicated in PolymiRTS for these 4 SNPs.

The screening of 26 cSNPs based on the putative function identified by four tools, is shown in Table 2. Four 3′UTR SNPs i.e., rs10845047, rs11053594, rs11053595, and rs11053597 predicted by all the tools were selected for further analysis. However, the findings of all the tools were not exactly overlapping for other SNPs. Therefore, SNPs whose functionality has been pre-suggested and putatively identified by one or more tools were selected for further analysis. These SNPs included rs11053593, rs7959451, rs7309123, rs2078178, and rs3901533. As a result, total 9 non-coding SNPs from intronic and 3′ UTR region of *CLEC7A* were sorted out from a pool of SNPs and recognized as fundamental variants. Out of the nine functionally identified casual variants of the present study, PCR-RFLP could be standardized for three SNPs rs11053593, rs11053597, and rs3901533 and thus validated.

**Table 2:** List of Conserved SNPs (cSNPs) screened based on all the tools used.

**Region**	**SNP ID**	**cSNPs**	**FuncPred**	**RegulomeDB score**	**PolymiRTS**	**Selected**
3′UTR	rs10845047	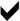	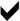	6	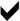	^*^
3′UTR	rs12304716	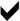	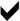	6	–	–
3′UTR	rs11053592	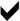	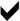	6	–	–
3′UTR	rs11053593^ 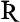 ^	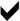	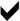	6	–	^*^
3′UTR	rs11053594	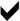	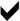	6	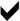	^*^
3′UTR	rs11053595	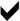	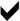	6	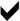	^*^
3′UTR	rs11053597	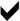	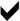	6	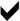	^*^
3′UTR	rs7959451^ 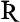 ^	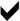	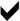	6	–	^*^
Intron5	rs7309123^ 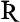 ^	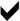	–	6	–	^*^
Intron5	rs11053602	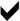	–	6	–	–
Intron5	rs58924693	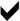	–	5	–	–
Intron5	rs5614055	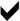	–	6	–	–
Intron5	rs59913193	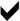	–	6	–	–
Intron5	rs7136680	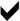	–	5	–	–
Intron5	rs4764272	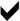	–	5	–	–
Intron5	rs11053608	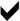	–	5	–	–
Intron5	rs7137840	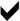	–	–	–	–
Intron4	rs2078178^ 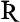 ^	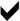	–	–	–	^*^
Intron4	rs5796380	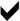	–	–	–	–
Intron4	rs3901533^ 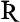 ^	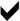	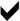	–	–	^*^
Intron4	rs11053613	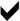	–	–	–	–
Intron4	rs3901532	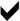	–	–	–	–
Intron3	rs11053617	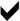	–	6	–	–
Intron3	rs4763446	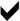	–	6	–	–
Intron2	rs35532667	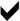	–	6	–	–
Intron2	rs7309870	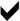	–	4	–	–

*cSNPs, conserved SNPs; 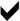, SNPs which affect function; –, SNPs which does not affect function*;

* *, prioritized for further studies*;

*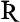, clinical significance has been reported (Sainz et al., 2012; Bacanu et al., 2014; Bennabi et al., 2015; Fischer et al., 2016; Overton et al., 2016, 2017)*.

### Influence of *CLEC7A* variants on the susceptibility to RVVI

Significantly low frequency of G allele of rs3901533 was found in RVVI cases than controls representing its role in protection against RVVI (*p* < 0.0001; OR = 0.43; 95% CI = 0.33–0.57) (Table 3). However, no difference was observed in allelic frequencies of rs11053593 and rs11053597 polymorphism of *CLEC7A* between RVVI cases and controls. For rs3901533 variant, the frequency of heterozygous (*p* = 0.004; OR = 0.53; 95% CI = 0.34–0.82) and homozygous GG genotype (*p* < 0.0001; OR = 0.24; 95% CI = 0.14–0.40) was considerably low in RVVI cases comparative to controls and thus defending against RVVI. However, no difference was observed in genotypic frequencies of rs11053593 and rs11053597 polymorphisms of *CLEC7A* between RVVI cases and controls (Table 4). All the hereditary models, excluding over-dominant, were significant for rs3901533 SNP. However, the log-additive model (*p* < 0.0001; OR = 0.49; 95% CI = 0.38–0.63) was found to be the best (AIC = 606.7, BIC = 619.1) among these, representing that if the risk for TG is *K*, the risk for TT will be *K*^2^ indicating that T allele in homozygous carrier had a higher risk of RVVI than its heterozygous carrier. However, no inheritance models were found to be significant for rs11053593 and rs11053597 polymorphisms.

**Table 3:** Allelic frequencies distribution of *CLEC7A* polymorphisms in RVVI cases and controls.

**Alleles**	**All subjects**	**RVVI cases**	**Controls**	**RVVI cases vs. controls**	
	**(*N* = 461)**	**(*N* = 258)**	**(*N* = 203)**	**OR (95% CI)**	***p*-value**
	**Freq (%)**	**Freq (%)**	**Freq (%)**		
rs11053593					
G	753 (82)	423 (82)	330 (81)	1	
A	169 (18)	93 (18)	76 (19)	0.95(0.68–1.33)	0.78
rs11053597					
G	751 (81)	421 (82)	330 (81)	1	
T	171 (19)	95 (18)	76 (19)	0.97 (0.70–1.36)	0.90
rs3901533					
T	526 (57)	340 (66)	186 (46)	1	
G	396 (43)	176 (34)	220 (54)	0.43 (0.33–0.57)	<0.0001^*^

*OR, odds ratio; CI, confidence intervals*;

* *indicates highly significant values (p ≤ 0.0001)*.

**Table 4:** Genotypic frequencies distribution of *CLEC7A* polymorphisms in RVVI cases and controls.

**Genetic Models**	**Genotype**	**RVVI Cases**	**Controls**	**OR (95% CI)**	***p*-value**	**AIC**	**BIC**
		**(*N* = 258)**	**(*N* = 203)**				
		**Freq (%)**	**Freq (%)**				
**rs11053593**							
Codominant	G/G	168 (65.1)	133 (65.5)	1.00	0.71	638.5	655
	G/A	87 (33.7)	64 (31.5)	1.08 (0.73–1.60)			
	A/A	3 (1.2)	6 (3.0)	0.39 (0.10–1.61)	0.19		
Dominant	G/G	168 (65.1)	133 (65.5)	1.00	0.93	638.5	650.9
	G/A-A/A	90 (34.9)	70 (34.5)	1.02 (0.69–1.50)			
Recessive	G/G-G/A	255 (98.8)	197 (97.0)	1.00	0.17	636.6	649
	A/A	3 (1.2)	6 (3.0)	0.39 (0.09–1.56)			
Overdominant	G/G-A/A	171 (66.3)	139 (68.5)	1.00	0.62	638.3	650.7
	G/A	87 (33.7)	64 (31.5)	1.11 (0.75–1.64)			
Log-additive	–	–	–	0.95 (0.67–1.35)	0.78	638.4	650.8
**rs11053597**							
Codominant	G/G	169 (65.5)	130 (64.0)	1.00	0.64	639.9	656.4
	G/T	83 (32.2)	70 (34.5)	0.91 (0.62–1.35)			
	T/T	6 (2.3)	3 (1.5)	1.54 (0.38–6.27)	0.54		
Dominant	G/G	169 (65.5)	130 (64.0)	1.00	0.74	638.4	650.8
	G/T-T/T	89 (34.5)	73 (36.0)	0.94 (0.64–1.38)			
Recessive	G/G-G/T	252 (97.7)	200 (98.5)	1.00	0.51	638.1	650.5
	T/T	6 (2.3)	3 (1.5)	1.59 (0.39–6.43)			
Overdominant	G/G-T/T	175 (67.8)	133 (65.5)	1.00	0.6	638.2	650.6
	G/T	83 (32.2)	70 (34.5)	0.90 (0.61–1.33)			
Log-additive	–	–	–	0.98 (0.69–1.39)	0.9	638.5	650.9
**rs3901533**							
Codominant	T/T	120 (46.5)	52 (25.6)	1.00	0.004^*^	608.6	625.1
	T/G	100 (38.8)	82 (40.4)	0.53 (0.34–0.82)			
	G/G	38 (14.7)	69 (34.0)	0.24 (0.14–0.40)	<0.0001^**^		
Dominant	T/T	120 (46.5)	52 (25.6)	1.00	<0.0001^**^	616.8	629.2
	T/G-G/G	138 (53.5)	151 (74.4)	0.40 (0.27–0.59)			
Recessive	T/T-T/G	220 (85.3)	134 (66.0)	1.00	<0.0001^**^	614.8	627.2
	G/G	38 (14.7)	69 (34.0)	0.33 (0.21–0.53)			
Overdominant	T/T-G/G	158 (61.2)	121 (59.6)	1.00	0.72	638.4	650.8
	T/G	100 (38.8)	82 (40.4)	0.93 (0.64–1.36)			
Log-additive	–	–	–	0.49 (0.38-0.63)	<0.0001^**^	**606.7**	**619.1**

*Akaike's Information Criterion (AIC) and Bayesian Information Criterion (BIC), Bold values indicate low AIC/BIC value*,

* *indicates significant values (p ≤ 0.01)*,

** *indicates significant values (p ≤ 0.001)*.

### Influence of *CLEC7A* variants on susceptibility to RVVI types

Comparisons of allelic and genotypic distribution were made between previously categorized RVVI cases (BV, VVC, and MI) and controls (Table 5; Kalia et al., 2015). For rs3901533 polymorphism, frequency of G allele and its homozygosity was appreciably low in BV (*p* = 0.0004; *p* = 0.0009 resp), VVC (*p* = 0.001; *p* = 0.002 resp) and MI cases (*p* = 0.0006; *p* = 0.002 resp) relative to controls and thus providing protection against RVVI types. However, heterozygosity for G allele was not found to be significantly different in either of the RVVI types comparative to controls. However, no difference was observed in allelic and genotypic frequencies of rs11053593 and rs11053597 polymorphism of *CLEC7A* between RVVI types and controls. Also, distribution of *CLEC7A* polymorphisms showed no difference between RVVI types (not shown in table). No homozygote for minor allele was observed in MI for rs11053593 polymorphism.

**Table 5:** Distribution and comparison of genotypic and allelic frequencies of *CLEC7A* polymorphisms in RVVI categories and controls.

	**No. (%) of controls**	**No. (%) of RVVI categories**			**Genotypic and allelic comparison**					
	**Controls (*N* = 203)**	**BV (*N* = 97)**	**VVC (*N* = 62)**	**MI (*N* = 41)**	**BV vs. Controls**		**VVC vs. controls**		**MI vs. controls**	
					**OR (95% CI)**	***p*-value**	**OR (95% CI)**	***p*-value**	**OR (95% CI)**	***p*-value**
**rs11053593**										
**Genotypes**										
GG	133 (65.5)	64 (65.90)	44 (70.96)	26 (63.41)	1		1		1	
GA	64 (31.5)	32 (32.90)	17 (27.41)	15 (36.58)	1.03 (0.61–1.74)	0.88	0.80 (0.42–1.51)	0.49	1.19 (0.59–2.41)	0.61
AA	6 (3.0)	1 (1.03)	1 (1.61)	0 (0)	0.34 (0.04–2.93)	0.33	0.50 (0.05–4.30)	0.53	–	NA
**Alleles**										
G	330 (81.0)	160 (82.47)	105 (84.60)	67 (81.70)	1		1		1	
A	76 (19.0)	34(17.52)	19 (15.32)	15 (18.29)	0.92 (0.59–1.44)	0.72	0.78 (0.45–1.35)	0.38	0.97 (0.52–1.79)	0.92
**rs11053597**										
**Genotypes**										
GG	130 (64.0)	63 (64.94)	43 (69.35)	30(73.17)	1		1		1	
GT	70 (34.5)	33 (34.02)	18 (29.03)	8 (19.5)	0.97 (0.58–1.62)	0.91	0.77 (0.41–1.44)	0.42	0.49 (0.21–1.13)	0.09
TT	3 (1.5)	1 (1.03)	1 (1.61)	3 (7.31)	0.68 (0.07–6.74)	0.74	1.00 (0.10–9.94)	0.99	4.33 (0.83–22.53)	0.08
**Alleles**										
G	330 (81.0)	159 (81.95)	104 (83.87)	68 (82.92)	1		1		1	
T	76 (19.0)	35 (18.04)	20 (16.12)	14 (17.07)	0.95 (0.61–1.48)	0.84	0.83 (0.48–1.43)	0.51	0.89 (0.47–1.67)	0.72
**rs3901533**										
**Genotypes**										
TT	52 (25.6)	37 (38.14)	26 (41.93)	18 (43.90)	1		1		1	
TG	82 (40.4)	45 (46.39)	26 (41.93)	19 (46.34)	0.77 (0.44–1.34)	0.36	0.63 (0.33–1.20)	0.16	0.66 (0.32–1.39)	0.28
GG	69 (34.0)	15 (15.46)	10 (16.12)	4 (9.75)	0.30 (0.15–0.61)	0.0009^**^	0.28 (0.12–0.65)	0.002^*^	0.16 (0.05–0.52)	0.002^*^
**Alleles**										
T	186 (46.0)	119 (61.34)	78 (62.90)	55 (67.07)	1		1		1	
G	220 (54.0)	75 (38.65)	46 (37.09)	27 (32.92)	0.53 (0.37–0.75)	0.0004^**^	0.49 (0.32–0.75)	0.001^**^	0.41 (0.25–0.68)	0.0006^**^

*OR, Odds Ratio; CI, Confidence Intervals; NA, not applicable*;

* *indicates high significant values (p ≤ 0.01)*,

** *indicates highly significant values (p ≤ 0.001)*.

### Linkage disequilibrium and haplotypes

**Figure 4:**
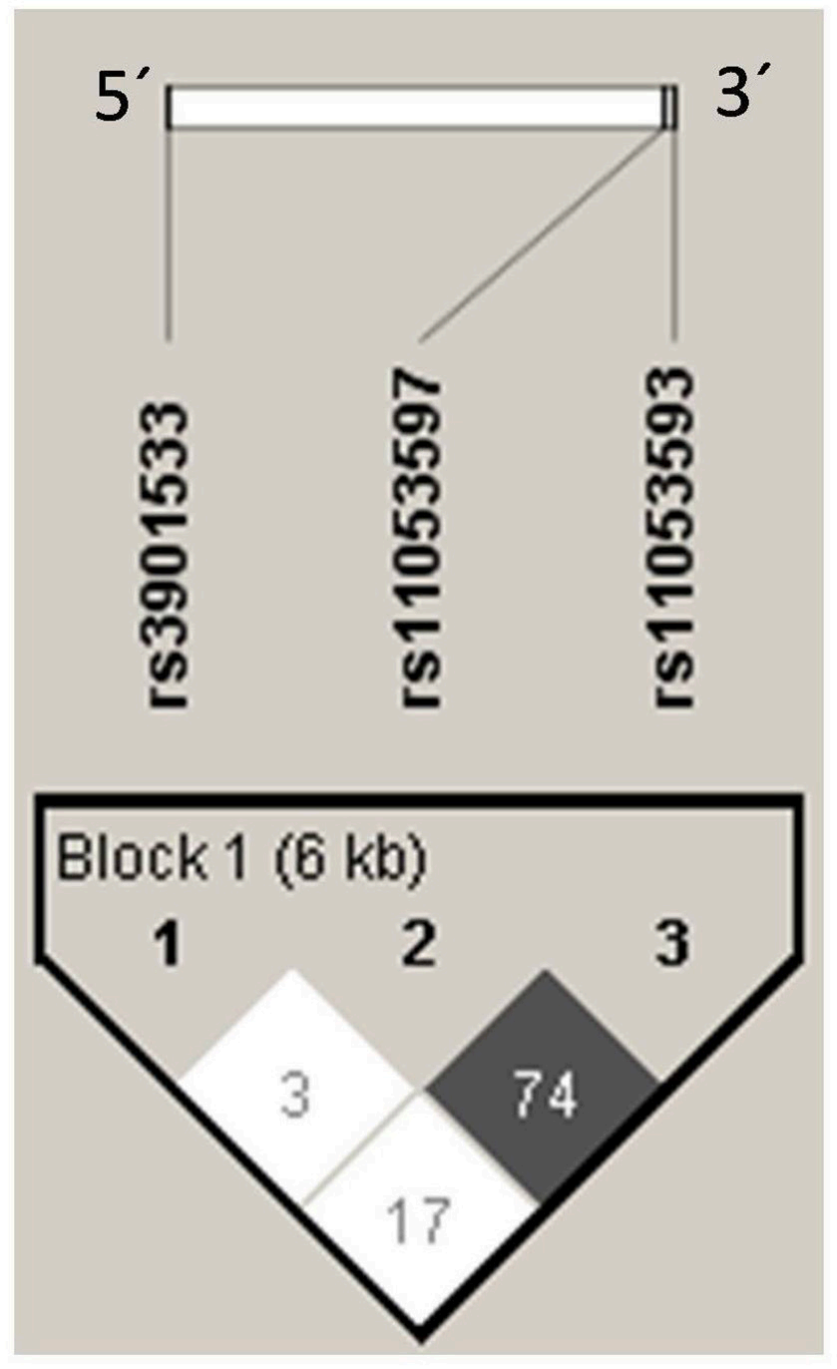
Linkage Disequilibrium plot of *CLEC7A* variants in all the participants. Numbers signifies the D' value expressed as percentile.

The Linkage disequilibrium (LD) analysis of the studied variants showed high LD between rs11053597 and rs11053593 (Figure 4). Both the SNPs i.e., rs11053597 and rs11053593 variants were not found to be in LD with rs3901533 variant. The *CLEC7A* haplotype (5′**→**3′) distribution was compared between groups (Table 6). These haplotypes represents SNP rs3901533, rs11053597 and rs11053593 in first, second and third position respectively. Global significant difference (*p* = 0.01) in haplotype frequencies was observed between cases and controls. A total of eight *CLEC7A* haplotypes were observed in the present study, of which TGG, GGG, and TTA were observed with frequency ≥0.05 in both cases and controls and were considered common haplotypes. While other haplotypes GTA, TGA, TTG, GTG, and GGA were observed with frequency ≤0.05 either in cases or controls were considered as rare haplotypes. Independent analysis of individual haplotypes indicated significantly low frequency of GGG haplotype in total RVVI cases (*p* < 0.0001), BV cases (*p* = 0.03), VVC cases (*p* = 0.03), and MI cases (*p* = 0.007) than controls. Similarly frequency of GTA haplotype was appreciably low in RVVI cases (*p* = 0.0003), BV cases (*p* = 0.002), VVC cases (*p* = 0.01), and MI cases (*P* = 0.04) than controls. Low frequency (*p* = 0.03) of GGA haplotype was observed in RVVI cases relative to controls.

**Table 6:** Distribution and comparison of *CLEC7A* haplotypes in cases and controls.

**Haplotypes (5′ → 3′)**	**No. (%) of controls**	**No. (%) of cases**				**Haplotype comparison**							
		**Total RVVI cases**	**Clinical categories of RVVI**			**RVVI vs. controls**		**BV vs. controls**		**VVC vs. controls**		**MI vs. controls**	
	**(*N* = 406)**	**(*N* = 516)**	**BV (*N* = 194)**	**VVC (*N* = 124)**	**MI (*N* = 82)**	**OR (95% CI)**	***p*-value**	**OR (95% CI)**	***p*-value**	**OR (95% CI)**	***p*-value**	**OR (95%CI)**	***p*-value**
TGG	143 (35.22)	258 (50.00)	86 (44.3)	58 (46.7)	40 (48.7)	1		1		1		1	
GGG	170 (41.87)	145 (28.10)	68 (35.0)	42 (32.8)	22 (26.8)	0.47 (0.34–0.63)	<0.0001^***^	0.66 (0.45–0.98)	0.03^*^	0.60 (0.38–0.96)	0.03^*^	0.46 (0.26–0.81)	0.007^**^
TTA	25 (6.15)	55 (10.65)	26 (13.4)	13 (10.4)	7 (8.5)	1.21 (0.72–2.04)	0.45	1.72 (0.93–3.18)	0.07	1.28 (0.61–2.67)	0.50	1.00 (0.40–2.48)	0.99
GTA	33 (8.12)	20 (3.87)	3 (1.5)	2 (1.6)	2 (2.4)	0.33 (0.18–0.60)	0.0003^***^	0.15 (0.04–0.50)	0.002^**^	0.14 (0.03–0.64)	0.01^**^	0.21 (0.04–0.94)	0.04^*^
TGA	11 (2.70)	15 (2.90)	5 (2.5)	4 (3.2)	6 (7.3)	0.75 (0.33–1.68)	0.49	0.75 (0.25–2.24)	0.61	0.89 (0.27–2.93)	0.85	1.95 (0.67–5.59)	0.21
TTG	7 (1.72)	11 (2.13)	2 (1.0)	3 (2.4)	2 (2.4)	0.87 (0.33–2.29)	0.78	0.47 (0.09–2.33)	0.36	1.05 (0.26–4.22)	0.93	1.02 (0.20–5.11)	0.97
GTG	10 (2.46)	9 (1.74)	4 (2.0)	2 (1.6)	3 (3.6)	0.49 (0.19–1.25)	0.13	0.66 (0.20–2.18)	0.50	0.49 (0.10 2.31)	0.37	1.07 (0.28–4.08)	0.91
GGA	7 (1.72)	3 (0.58)	0 (0.0)	0 (0.0)	0 (0.0)	0.23 (0.06–0.93)	0.03^*^	**–**	NA	–	NA	–	NA

*Global p-value for case/control haplotype association was 0.01; NA, not applicable*.

* *p ≤ 0.05*;

** *p ≤ 0.01*;

*** *p ≤ 0.001*.

### Serum levels of dectin-1 in RVVI cases and healthy controls

**Figure 5:**
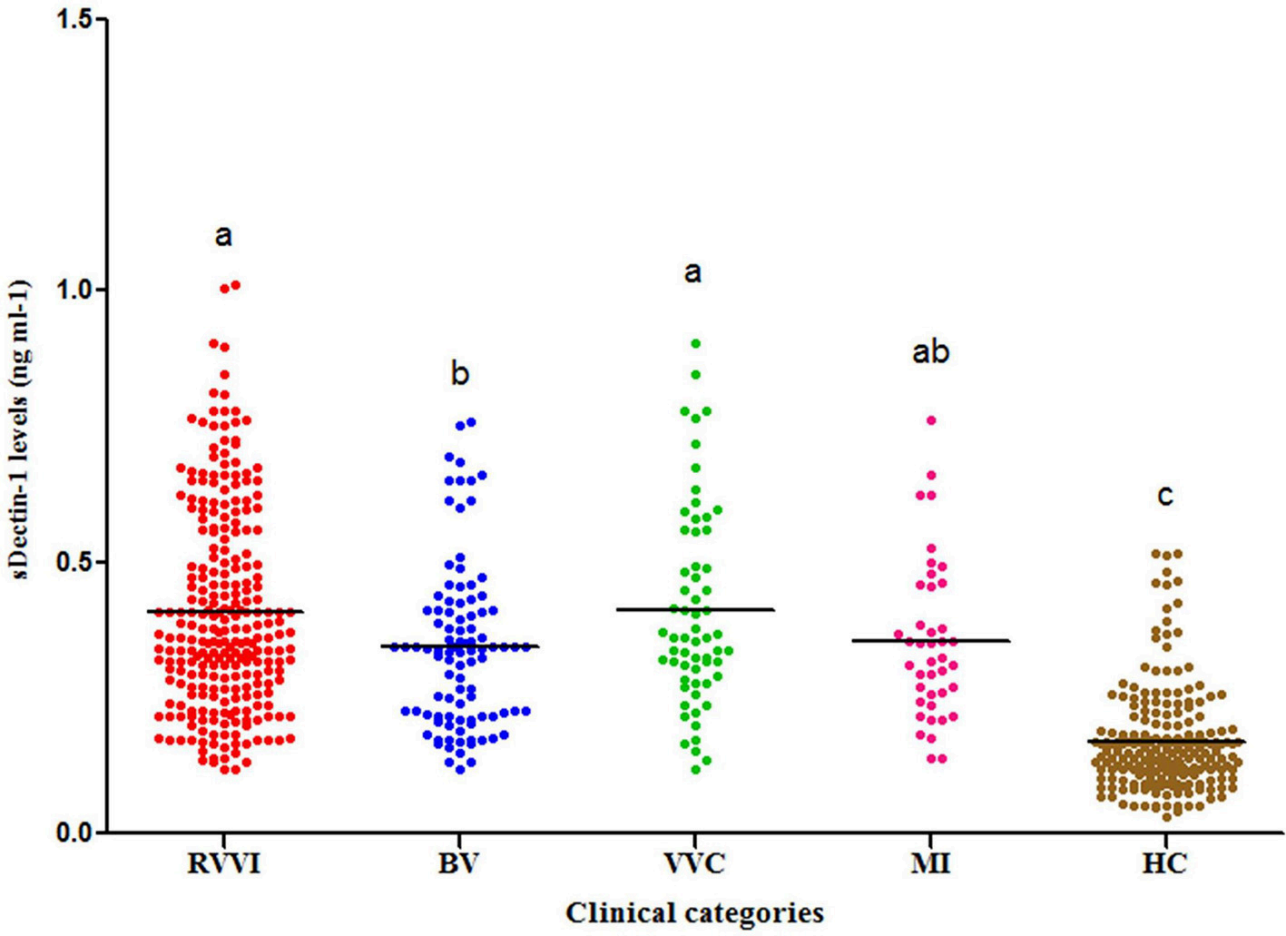
Serum Dectin-1 levels in different categories of cases and healthy controls (HC). Dot plot showing distribution of sDectin-1 levels in cases and controls. Horizontal line represents the mean value. One Way ANOVA followed by *Post hoc* Tukey test. Categories with different letters are significantly different from each other at *p* < 0.05.

Significantly high sdectin-1 levels were observed in RVVI cases and its subtypes BV, VVC, and MI relative to controls (Figure 5). Also, high sDectin-1 levels were observed in RVVI and VVC cases than BV (*p* < 0.05).

### Influence of *CLEC7A* genotypes on sDectin-1 levels

**Figure 6:**
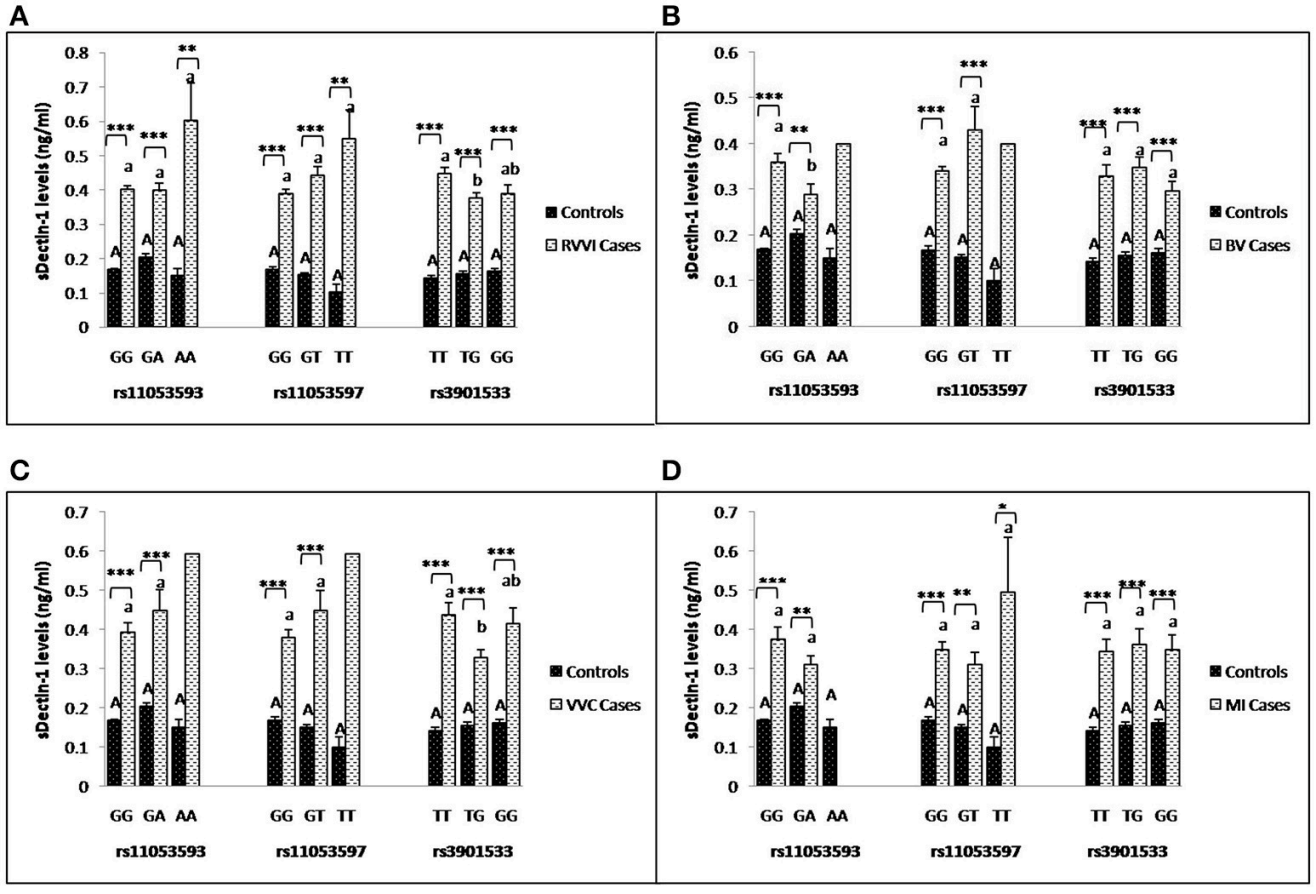
Comparison of sDectin-1 levels in different *CLEC7A* genotypes. sdectin-1 levels (mean±standard error) segregation based on different genotypes of studied *CLEC7A* variants in controls with RVVI **(A)** and controls with RVVI types including BV **(B)** VVC **(C)** and MI **(D)**. Asterisks corresponds to Student's *t*-test comparing cases with respective controls (^*^p < 0.05; ^**^p < 0.01; ^***^p < 0.001). Letters corresponds to Tukey's multiple comparison test, where capital letters compare genotypic sdectin-1 levels of single polymorphism within controls and small letters compare genotypic sdectin-1 levels of single polymorphism within cases. Genotypes with different letters are significantly different from each other at *p* < 0.05. Genotypes with no letters are excluded from statistical analysis due to their low number observed in present study.

Segregation of sdectin-1 levels were made on the basis of *CLEC7A* genotypes in different cases and controls (Figures 6A–D). It was found that different genotypes of individual SNP showed no significant difference in sdectin-1 levels within controls. However, TT genotype of rs3901533 was contributing significantly high sDectin-1 levels than TG genotype in RVVI and VVC cases. For rs11053593, sdectin-1 levels for GG genotypes were significantly high than GA genotype in BV cases. No other difference in genotypic sdectin-1 levels were observed within cases. Also, significantly high sdectin-1 levels were found in genotypes of RVVI and its types relative to respective control genotypes in various *CLEC7A* polymorphisms.

### Influence of *CLEC7A* haplotypes on sDectin-1 levels

**Figure 7:**
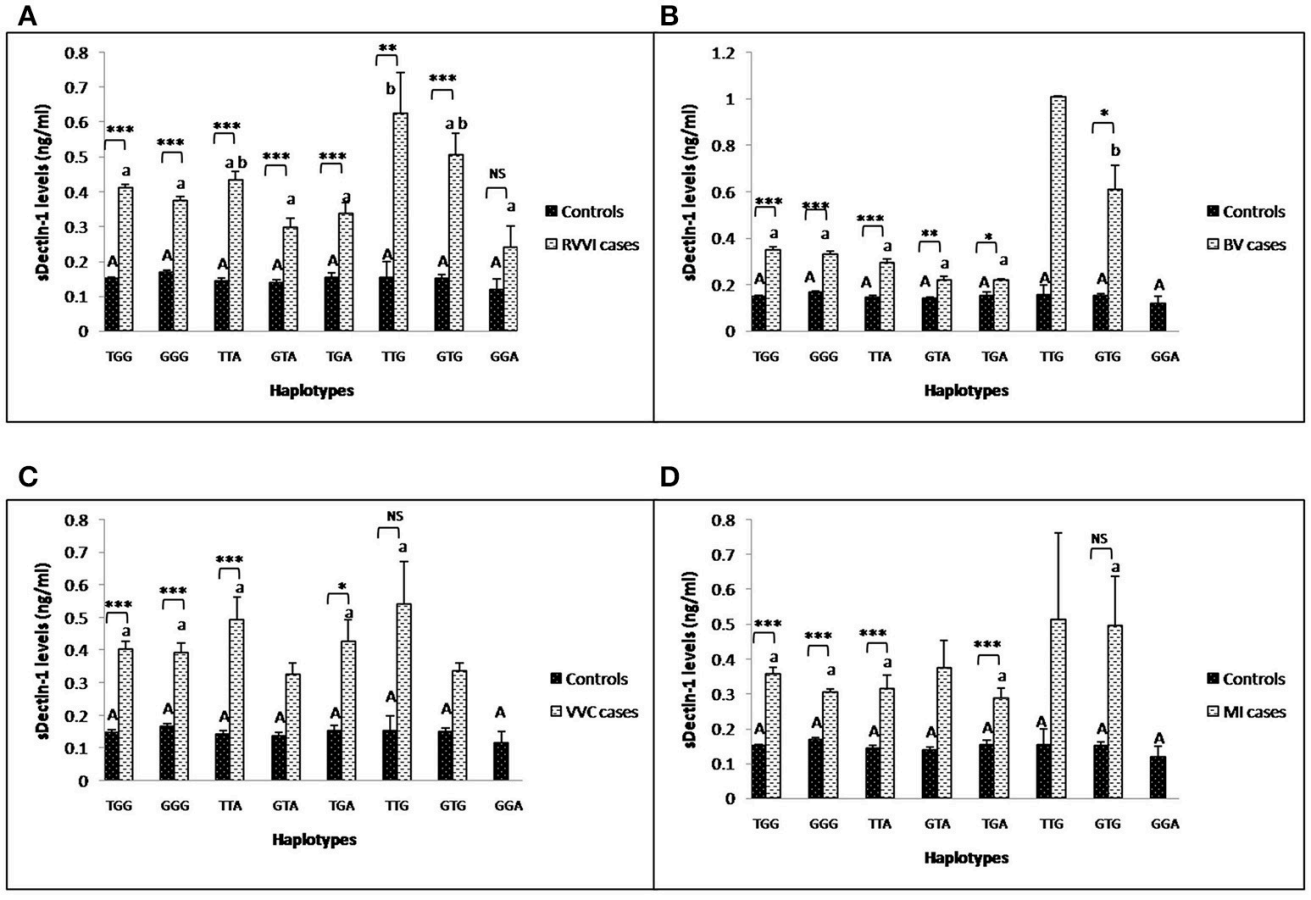
Comparison of sDectin-1 levels in different *CLEC7A* haplotypes. sdectin-1 levels (mean±standard error) segregation based on different haplotypes of studied *CLEC7A* variants in controls with RVVI **(A)** and controls with RVVI types including BV **(B)** VVC **(C)** and MI **(D)**. Asterisks corresponds to Student's *t*-test comparing cases with respective controls (^*^p < 0.05; ^**^p < 0.01; ^***^p < 0.001). Letters corresponds to Tukey's multiple comparison test, where capital letters compare haplotypic sdectin-1 levels of single polymorphism within controls and small letters compare haplotypic sdectin-1 levels of single polymorphism within cases. Haplotypes with different letters are significantly different from each other at *p* < 0.05. Haplotypes with no letters are excluded from statistical analysis due to their low number observed in present study.

Mean levels of sDectin-1 of individual haplotypes were also studied in different cases groups and controls (Figures 7A–D). sDectin-1 levels of haplotypes showed no difference within control. In RVVI cases, TTG haplotype was contributing significant high levels than all the other haplotypes of RVVI cases except TTA and GTG. In BV cases, significantly high dectin-1 levels were observed in GTG haplotype than all the other haplotypes. No significant difference in haplotypic sDectin-1 levels was observed within VVC and MI cases. Also, significant difference was observed in haplotypic sDectin-1 levels of RVVI cases and its types with respective controls haplotypes.

### Correlation between sDectin-1 and sMBL levels

**Figure 8:**
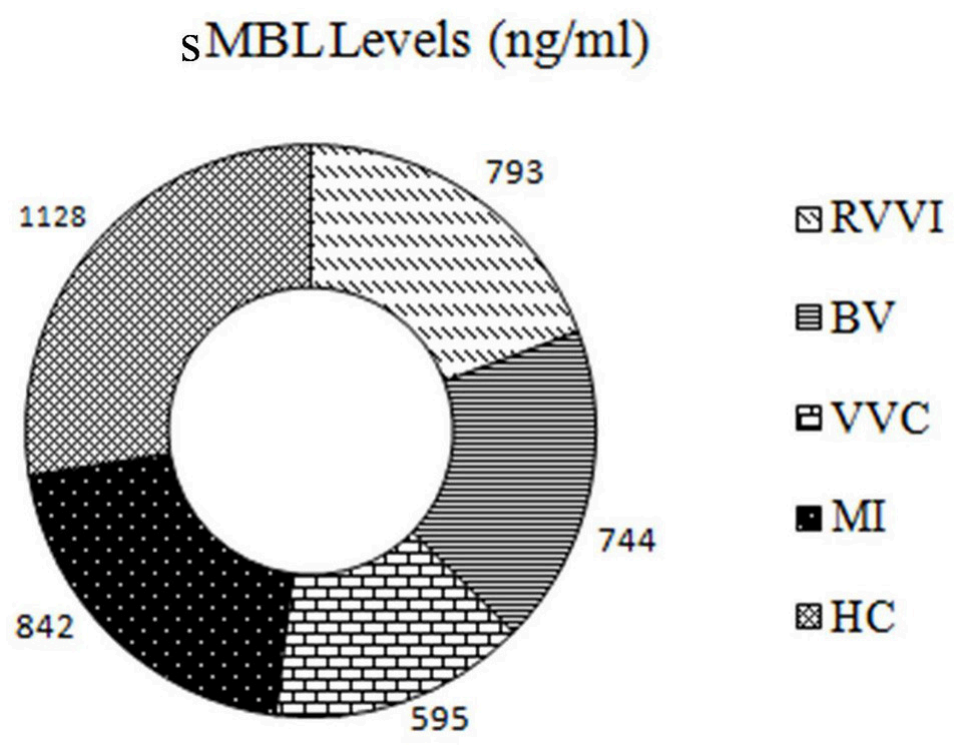
The graphic of sMBL levels in all the studied groups: The donut representing sMBL levels in cases and controls. Figure reproduced from data of our previous report (Kalia et al., 2017).

Distribution of sMBL levels in RVVI cases and healthy controls have been reported previously, with significantly low sMBL levels in RVVI cases and its subtypes relative to controls (Figure 8; Kalia et al., 2017). The correlation analysis between sDectin-1 and sMBL levels showed no significant correlation in all the studied groups including RVVI (*p* = 0.423; *r* = −0.012), BV (*p* = 0.330; *r* = 0.045), VVC (*p* = 0.062; *r* = −0.198), MI (*p* = 0.425; *r* = 0.031) and controls (*p* = 0.199; *r* = −0.060).

### Influence of *CLEC7A* genotypes on sMBL levels

**Figure 9:**
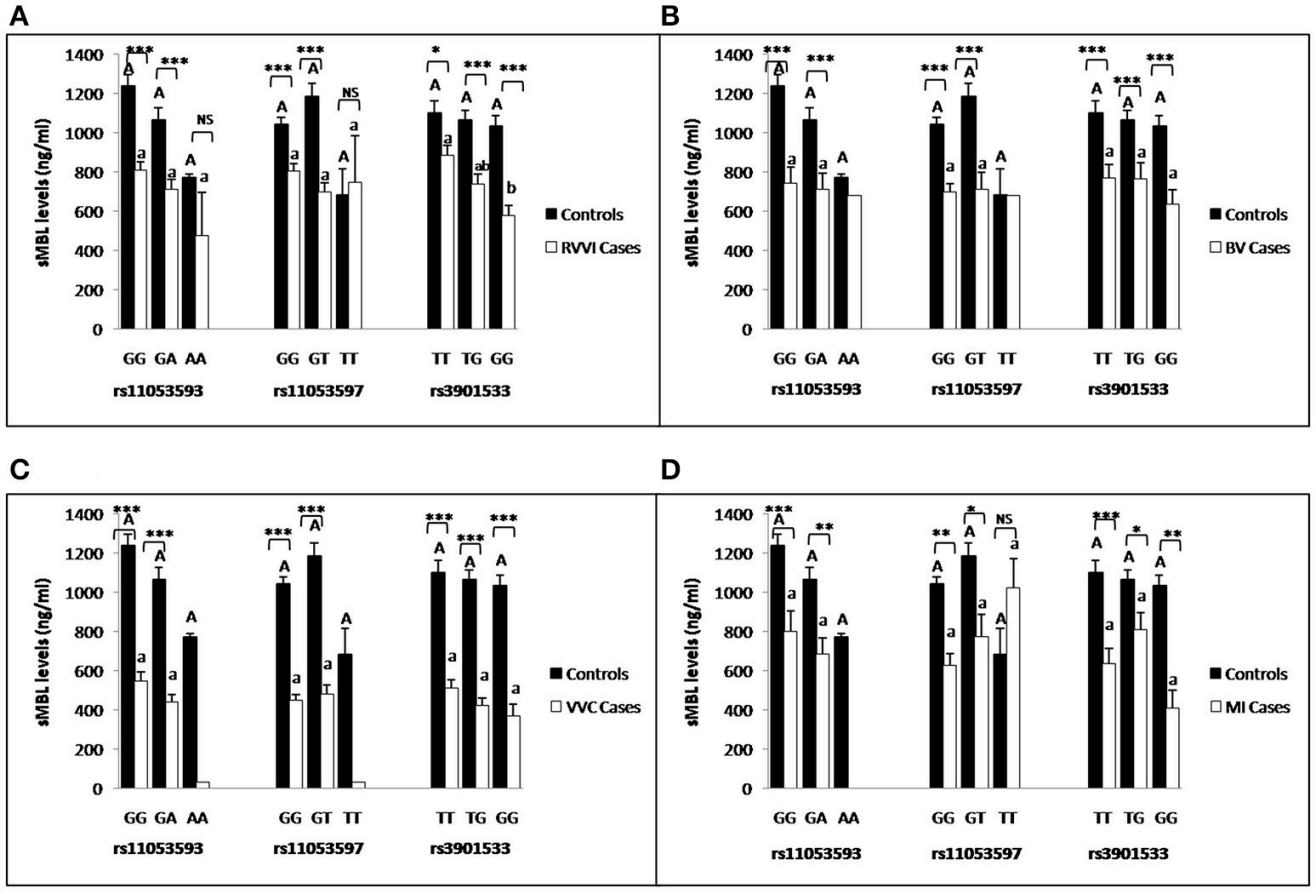
Comparison of sMBL levels in different *CLEC7A* genotypes. sMBL levels (mean±standard error) segregation based on different genotypes of studied *CLEC7A* variants in controls with RVVI **(A)** and controls with RVVI types including BV **(B)** VVC **(C)** and MI **(D)**. Asterisks corresponds to Student's *t*-test comparing cases with respective controls (^*^p < 0.05; ^**^p < 0.01; ^***^p < 0.001). Letters corresponds to Tukey's multiple comparison test, where capital letters compare genotypic sMBL levels of single polymorphism within controls and small letters compare genotypic sMBL levels of single Polymorphism within cases. Genotypes with different letters are significantly different from each other at *p* < 0.05. Genotypes with no letters are excluded from statistical analysis due to their low number observed in present study.

Segregation of sMBL levels were made on the basis of *CLEC7A* genotypes in different cases and controls (Figures 9A–D). For rs3901533 GG genotype was contributing significantly low sMBL levels than TT genotype in RVVI cases. However no other difference was found in genotypic sMBL levels of *CLEC7A* polymorphisms in RVVI categories and in controls. Also, RVVI cases and its types showed significantly low genotypic sMBL levels as compared to respective genotypes in controls for various *CLEC7A* polymorphisms.

### Influence of *CLEC7A* haplotypes on sMBL levels

**Figure 10:**
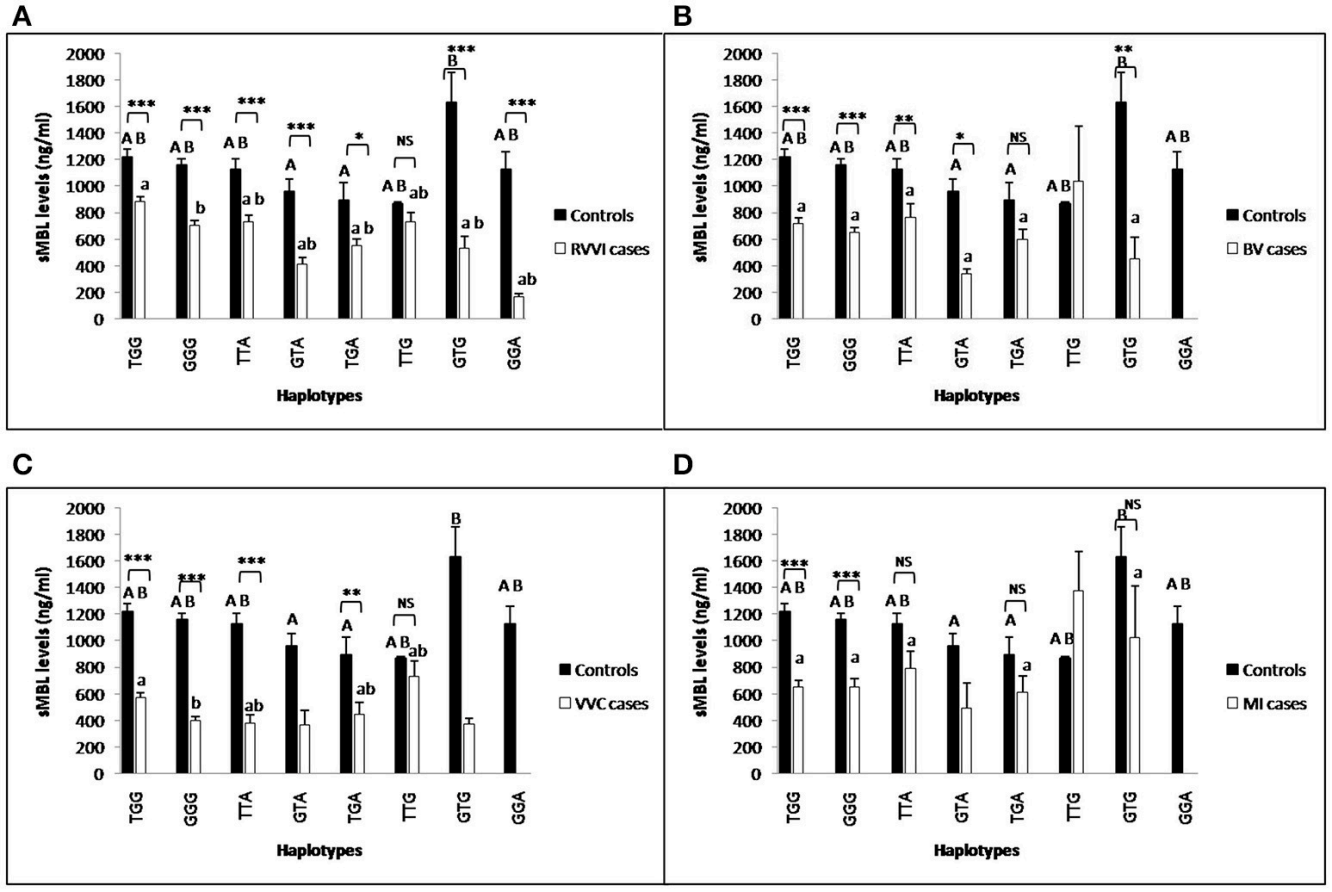
Comparison of sMBL levels in different *CLEC7A* haplotypes. sMBL levels (mean±standard error) segregation based on different haplotypes of studied *CLEC7A* variants in controls with RVVI **(A)** and controls with RVVI types including BV **(B)** VVC **(C)**, and MI **(D)**. Asterisks corresponds to Student's *t*-test comparing cases with respective controls (^*^p < 0.05; ^**^p < 0.01; ^***^p < 0.001). Letters corresponds to Tukey's multiple comparison test, where capital letters compare haplotypic sMBL levels of single polymorphism within controls and small letters compare haplotypic sMBL levels of single polymorphism within cases. Haplotypes with different letters are significantly different from each other at *p* < 0.05. Haplotypes with no letters are excluded from statistical analysis due to their low number observed in present study.

Individual *CLEC7A* haplotypes were also studied for the distribution of mean sMBL levels in cases and control groups (Figures 10A–D). In controls, GTA and TGA haplotypes were contributing significant low levels than GTG haplotype, while, no other difference was observed. In RVVI and VVC cases, GGG haplotype was contributing significant low levels than TGG haplotype, while no other differences were observed. Also, various haplotypes of RVVI and its categories were contributing significantly low sMBL levels than their respective haplotype in controls.

## Discussion

Recurrent vulvovaginal infections are the commonly reported microbiological syndrome affecting millions of women globally. Since many women develop RVVI without any known predisposing factor, there may be genetic predisposition that could act as a risk factor for RVVI in women (Bleicher and Stockdale, 2015; Sobel, 2016). Recently, we found that genetic polymorphisms in innate immune gene, Mannose binding Lectin (*MBL2*) and its low serum levels contributed as risk factors toward RVVI (Kalia et al., 2017). However, our previous findings also suggested the probable contribution of several other host genes that may collectively confer risk of RVVI, further implying RVVI as a multifactorial/polygenic phenotype. Dectin-1 is another such important molecule of innate immunity that is best known for its role in antifungal defense and in providing defense against several other pathogens (Ferwerda et al., 2009; Drummond and Brown, 2013; Lefèvre et al., 2013; Heyl et al., 2014). Therefore, exploring whether the *CLEC7A* polymorphisms affects susceptibility to RVVI or not, is the purpose of the present study.

There are total 1179 SNPs in dbSNP database of human *CLEC7A* that have been identified. However, majority of these SNPs are still not functionally recognized. Therefore effort was made to haul out the most likely functional variants of *CLEC7A* using *in silico* analysis from the SNPs that may not have any function or of very low frequency (Bhatti et al., 2006; Johnson, 2009; Li and Wei, 2015). In order to identify, putative functional significance of given SNPs, various complementary bioinformatics tools are used in the present study. This is because, a complete depiction of SNP functions, cannot be obtained by using single bioinformatics tool. Therefore, the tools used in the current analysis include SNPinfo, Ensembl, regulomeDB, and poylmiRTS. In order to avoid frequent pitfalls that occurs while carrying out an *in silico* analysis some essential features were considered. First is, verifying the consistency of a given SNP by considering its validation status (Fredman et al., 2002). Second is, removing false-positive prophecy that occurs due to *in silico* tools, this is done by considering conserved SNPs only (Cartegni et al., 2003; Yeo and Burge, 2004). The third very important feature is the use of MAF. MAF is inversely proportional to the sample size of the study needed to detect risk allele (Grover et al., 2007). This sample size further has a direct relationship with power of study to detect statistical significance. Therefore, MAF cut-off of ≥0.10 was considered for the present analysis. The fourth important feature was the use of literary evidences for the SNPs for which results from different tools were not directly overlapping. Thus by considering these important characteristics in mind and based on the results of tools used, nine non-coding SNPs of *CLEC7A* were found to be functionally important for disease association studies.

A very scanty data is available regarding role of these selected SNPs of *CLEC7A*. Of these, rs7309123 and rs3901533 SNPs were found to be associated with increased susceptibility to invasive pulmonary aspergillosis (IPA) infection and rs7309123 with developing invasive fungal diseases in patient with Acute Myeloid Leukemia (AML) as well as severe asthma with fungal sensitization (SAFS) (Sainz et al., 2012; Fischer et al., 2016; Overton et al., 2017). rs2078178 influences the autism spectrum disorders (ASD) risk (Bennabi et al., 2015). These reports validate and complement the findings of the current study. However, the role of rs3901533 in AML and fungal keratitis, rs7959451 in SAFS, rs7309123 in allergic bronchopulmonary aspergillosis (ABPA) in asthma were evaluated but no association were found with the diseases studied perhaps due to genetic heterogeneity found in different ethnicities or due to disease specificity (Qu et al., 2015; Fischer et al., 2016; Overton et al., 2016, 2017). Moreover, no clinical significance of rs10845047, rs11053594, rs11053595, and rs11053597 is recognized till date. Therefore, more studies in clinical settings are needed to verify these nine functionally important SNPs reported in the present study. Therefore, effort was made in this study to investigate their role in RVVI.

As most of the SNPs that were predicted to be functional by *in silico* analysis are new and those which were reported are genotyped solely based on direct sequencing or other technique like pyro-sequencing, TaqMan assay and so forth that requires the purchase of expensive equipment or chemicals. Therefore, effort was made in this study to standardize a straight forward method for genotyping/validating these polymorphisms i.e., polymerase chain reaction-restriction fragment length polymorphism (PCR–RFLP) which is inexpensive, simple and convenient method for SNP genotyping (Ota et al., 2007). Fortunately, we were able to design PCR-RFLP for three SNPs i.e., rs11053593, rs11053597, and rs3901533 that can be confidently used as a cheaper alternative genotyping method for these sites. While other target SNPs were not found to be suitable for commercial restriction enzymes, or inversely, the sequence of *CLEC7A* for particular SNP have numerous restriction sites for a given restriction enzyme thus limiting the use of this technique for these SNPs. Therefore, the aforesaid 3 *CLEC7A* variants are the focus of the present study. Furthermore, the role of these variants in RVVI and its types has not been investigated earlier, making the current analysis first approach toward it. Also, the frequency distribution of 3 *CLEC7A* variants is reported for the first time by the present study in Indian population. rs11053593_A, rs11053597_T, and rs3901533_G were found to be minor alleles with MAF 0.18, 0.19 and 0.43 respectively. This is in consonance with overall MAF and MAF of individual populations of 1000 genome project for these SNPs (Table S4). However, a skew in the frequency of rs3901533 SNP was observed in American and European population leading to an excess of rare variant which is in contrast to the present study.

*CLEC7A* rs3901533 polymorphism was found to be modulating the susceptibility for RVVI and its types. Its minor allele and its homozygosity were significantly lowering the risk of developing RVVI and its types, whereas homozygous carriers of its major allele were at risk. No study has assessed the role of rs3901533 polymorphism in relation to RVVI and its types. However, these results are in agreement with previous study reporting the significant association of this SNP with increased risk of IPA infection (Sainz et al., 2012). But in contrast to the studies that have found no association of *CLEC7A* rs3901533 with fungal keratitis and AML (Qu et al., 2015; Fischer et al., 2016). The above inconsistency in results might stem from genetic heterogeneity found in different ethnicities or due to disease specificity, thus must be evaluated in different population. Stratification based on *CLEC7A* haplotypes indicated that GGG (rs3901533_G) and GTA (rs3901533_G) haplotypes have provided significant protection against RVVI (OR; 0.47, 0.33 resp.), BV (OR; 0.66, 0.15 resp.), VVC (OR; 0.60, 0.14 resp.) and MI (OR; 0.46, 0.21 resp.). These findings suggested minor allele of rs3901533 as an important marker for providing defense against RVVI and its types.

**Figure 11:**
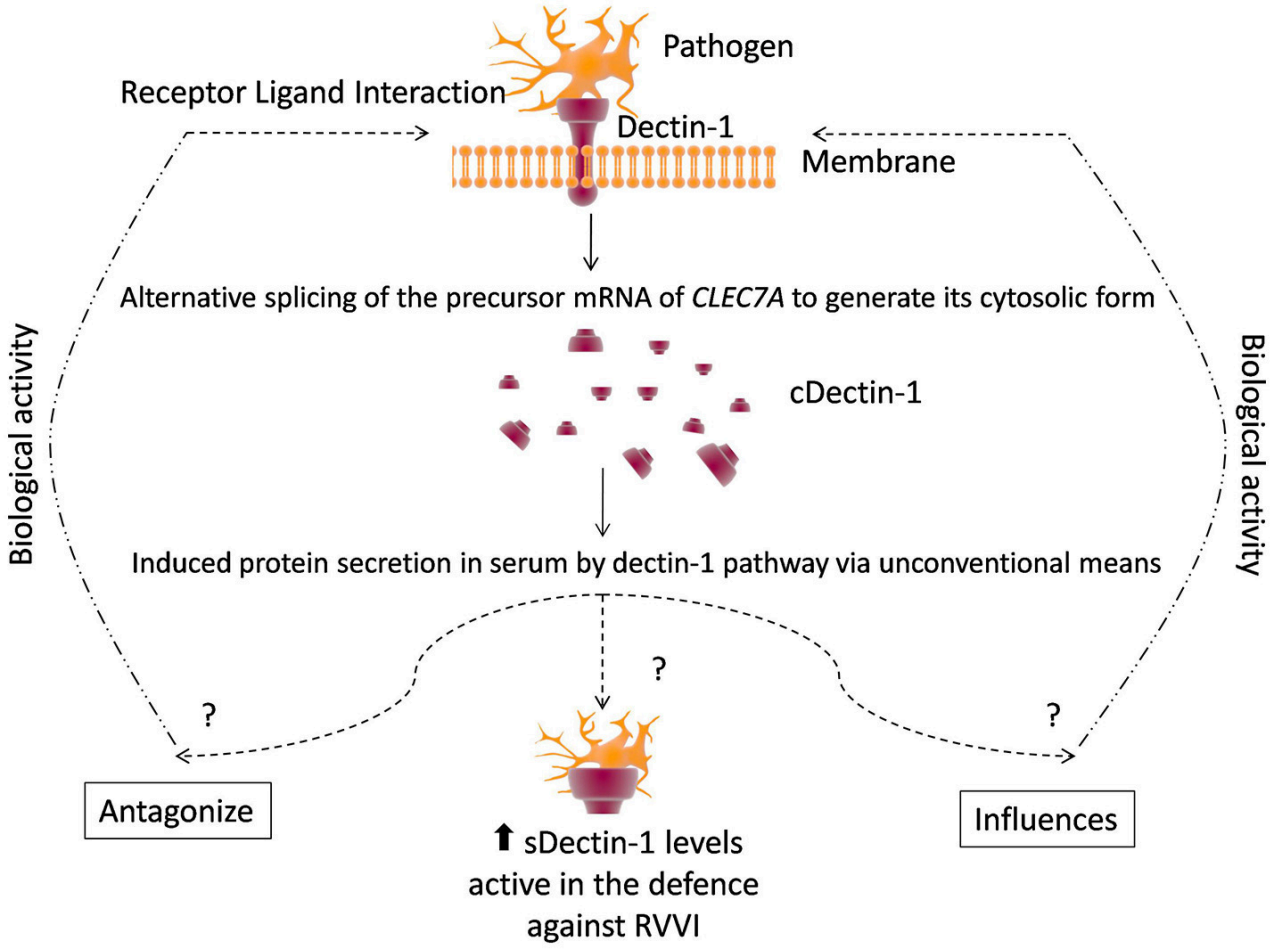
A conceptual model of defense by dectin-1 and regulation by its circulatory form. See text for citations.

Significantly high sdectin-1 levels were detected in RVVI cases and its various categories than controls, suggesting high sdectin-1 levels as a disease specifier for RVVI and its types. Among RVVI types, VVC showed significantly high sdectin-1 levels than BV and MI. This is in consonance with an earlier study that showed increased intracellular expression of dectin-1 in neutrophils after *C. albicans* stimulation (Li D. et al., 2012). As dectin-1 pathway activates unconventional protein secretion, it may effectively induce secretion of its soluble form by unconventional means in serum (Öhman et al., 2014). Higher organisms employed an influential regulatory system i.e., alternative splicing for producing distinct functional proteins form same precursor mRNA that significantly explains proteomic complexity (Mummidi et al., 2001). These different functional proteins from a single gene either influence or cross-regulate the performance of the other isoforms. Some supporting examples includes, two isoforms of mouse dectin-1, that are functionally different in identifying zymosan and producing TNF-alpha and the same properties are also shared by corresponding human dectin-1 isoforms (Heinsbroek et al., 2006). Another example, where one spliced isoform influences the function of another original full length isoform is CD40 (Tone et al., 2001). Similarly, whether this soluble form of dectin-1 up-regulates or down-regulates the expression of other dectin-1 isoforms, particularly its transmembrane form, remains elusive (Figure 11).

Association between *CLEC7A* polymorphisms and sdectin-1 levels were also investigated in the present study. Significantly high sdectin-1 levels were found in cases than controls for the corresponding genotypes and haplotypes, suggesting the active role played by dectin-1 in defense against pathogens causing RVVI. In RVVI and VVC, TG genotype contributed low sdectin-1 levels than wild TT genotype. In RVVI, TTG haplotype was contributing significantly high levels than all the other haplotypes of RVVI cases except TTA and GTG. In BV, GTG haplotype was contributing significant high levels than all the other haplotypes of BV cases. These observations suggest that irrespective of the observed haplotype region, there are other variants that may be involved in regulating sdectin-1 levels and hence cannot ignore.

Influence of the *CLEC7A* polymorphisms on sMBL levels, were also assessed. Considerably low sMBL levels were found in cases comparative to controls for the corresponding *CLEC7A* genotypes and haplotypes. Also in RVVI cases, GG genotype of rs3901533 contributed significantly low sMBL levels than TT genotype. In RVVI and VVC cases, GGG haplotype (rs3901533_G) was contributing significantly low sMBL levels than TGG (rs3901533_T). This shows that, rs3901533 SNP may be modulating sMBL levels via altering dectin-1 pathway that induces collaborative inflammatory responses and defense against RVVI. However, our other findings showed no significant correlation between sDectin-1 and sMBL levels in RVVI cases and controls. This further suggests that the association found between *CLEC7A* polymorphisms and sMBL levels may simply be due to co-activation of two PRRs against the same pathogenic stimuli i.e., RVVI. The only study that so far collaborate the two PRRs has showed increased intracellular levels of dectin-1 by MBL-arbitrated opsonophagocytosis of pathogens (Li D. et al., 2012). Thus, further studies are needed to confirm the complex relationship between these two CLRs in RVVI.

In the present study, allelic and genotypic frequencies of dectin-1 polymorphisms are in Hardy Weinberg Equilibrium (HWE) except for rs3901533 (*p* < 0.0001). This deviation from HWE is accredited to selection pressure or population admixture (Hosking et al., 2004). The reason may possibly be participants belonging to same geological area, hospital based study or due to infectious diseases (Miller, 1999; Ghosh, 2008; Fumagalli et al., 2009).

In conclusion, the present analysis demonstrated that 9 putative functional SNPs of *CLEC7A*. Many of these variants have not been explored much till date. The frequency distribution of three *CLEC7A* variants i.e., rs11053593, rs11053597, and rs3901533 are reported for the first time in a representative sample of Indian population. The method used for the genotyping of these SNPs constitutes a simple and reliable assay which is compatible to facilities found in most of the laboratories. Our study found novel associations of *CLEC7A* rs3901533 polymorphism and high sDectin-1 levels with RVVI and its types. The same polymorphism was also found to be associated with sMBL levels that require further studies to confirm the complex relationship, as the association may simply be due to same pathogenic stimuli. Thus, aforementioned novel associations can be used for examining women with RVVI for better diagnosis and treatments. These are the preliminary findings and future investigations, to assess the contribution of additional *CLEC7A* variants in RVVI, are required using reliable methods. Finally, functional studies are warranted to define the implication of *CLEC7A* SNPs and haplotypes on gene expression, mRNA stability as well as structural and physiological aspects of dectin-1. Also, genotype-phenotype correlations of the current analysis recommended the probable contribution of other host genes that may collectively confer risk of RVVI, further implying RVVI as a polygenic phenotype. These genes must be explored to explain the genetic factors that impact woman's tendency to develop RVVI.

## Author contributions

NK reviewed the literature, was involved in design, performing experiments, analysis, interpretation, and drafted the manuscript. SS participated in sample collection and RVVI diagnosis. MK, JS, and SS contributed in the experimental design, data analysis, manuscript editing and supervision. All authors read and approved the final manuscript.

### Conflict of interest statement

The authors declare that the research was conducted in the absence of any commercial or financial relationships that could be construed as a potential conflict of interest. The reviewer HF and handling Editor declared their shared affiliation.
