# Supplementary material for: A Comprehensive in Silico Analysis of Regulatory SNPs of Human CLEC7A Gene and Its Validation as Genotypic and Phenotypic Disease Marker in Recurrent Vulvovaginal Infections
Source: Front Cell Infect Microbiol. 2018 Mar 20;8:65. doi: 10.3389/fcimb.2018.00065 (PMC5869923; doi:10.3389/fcimb.2018.00065)
Supplement: Supplementary file 1 [file Table1.DOCX]

**Table S1** List of conserved non-coding SNPs predicted by FuncPred

| REGION | SNP ID | Chr. Position | Splicing | miRNA |
| --- | --- | --- | --- | --- |
| 3´UTR | rs10845047 | 10116823 | - | ✔ |
| 3´UTR | rs12304716 | 10116845 | ✔ | ✔ |
| 3´UTR | rs11053592 | 10117575 | - | ✔ |
| 3´UTR | rs11053593 | 10118214 | - | ✔ |
| 3´UTR | rs11053594 | 10118222 | - | ✔ |
| 3´UTR | rs11053595 | 10118223 | - | ✔ |
| 3´UTR | rs11053597 | 10118339 | - | ✔ |
| 3´UTR | rs7959451 | 10118456 | - | ✔ |
| Intron | rs3901533 | 10124484 | - | ✔ |

✔: SNPs which affect function; - : SNPs which does not affect function.
