# Supplementary material for: A Comprehensive in Silico Analysis of Regulatory SNPs of Human CLEC7A Gene and Its Validation as Genotypic and Phenotypic Disease Marker in Recurrent Vulvovaginal Infections
Source: Front Cell Infect Microbiol. 2018 Mar 20;8:65. doi: 10.3389/fcimb.2018.00065 (PMC5869923; doi:10.3389/fcimb.2018.00065)
Supplement: Supplementary file 2 [file Table2.DOCX]

**Table S2** List of SNPs predicted by regulomeDB score

| dbSNP ID | | RDB score | | Category | Description |  |
| --- | --- | --- | --- | --- | --- | --- |
|  |  | |  |  |  |  |
|  |  | |  |  |  |  |
| rs7309870 |  | | 4 | Minimal binding evidence | TF binding + DNase peak |  |
|  |  | |  | Minimal binding evidence |  |  |
| rs11053608 |  | | 5 |  | TF binding or DNase peak |  |
| rs4764272 |  | |  |  |  |  |
| rs58924693 |  | |  |  |  |  |
| rs7136680 |  | |  |  |  |  |
|  |  | |  |  |  |  |
| rs10845047 |  | | 6 | Minimal binding evidence | Motif hit |  |
| rs11053592 |  | |  |  |  |  |
| rs11053593 |  | |  |  |  |  |
| rs11053594  rs11053595  rs11053597 |  | |  |  |  |  |
| rs11053602 |  | |  |  |  |  |
| rs11053617 |  | |  |  |  |  |
| rs12304716 |  | |  |  |  |  |
| rs35532667 |  | |  |  |  |  |
| rs4763446 |  | |  |  |  |  |
| rs56140555 |  | |  |  |  |  |
| rs59913193 |  | |  |  |  |  |
| rs7309123 |  | |  |  |  |  |
| rs7959451 |  | |  |  |  |  |
|  | | | | | | |

RDB - RegulomeDB, TF – transcription factor
