# Supplementary material for: A Comprehensive in Silico Analysis of Regulatory SNPs of Human CLEC7A Gene and Its Validation as Genotypic and Phenotypic Disease Marker in Recurrent Vulvovaginal Infections
Source: Front Cell Infect Microbiol. 2018 Mar 20;8:65. doi: 10.3389/fcimb.2018.00065 (PMC5869923; doi:10.3389/fcimb.2018.00065)
Supplement: Supplementary file 3 [file Table3.DOCX]

**Table S3** List of 3´UTR SNPs analyzed by PolymiRTS.

| dbSNP ID | Alleles | | Predicted miRNA motif |
| --- | --- | --- | --- |
|  |  | |  |
| [rs10845047](http://www.ncbi.nlm.nih.gov/SNP/snp_ref.cgi?rs=rs10845047) | A | | [hsa-miR-4803](http://www.mirbase.org/cgi-bin/query.pl?terms=hsa-miR-4803) |
|  | T | | [hsa-miR-3613-3p](http://www.mirbase.org/cgi-bin/query.pl?terms=hsa-miR-3613-3p)  [hsa-miR-495-3p](http://www.mirbase.org/cgi-bin/query.pl?terms=hsa-miR-495-3p)  [hsa-miR-5688](http://www.mirbase.org/cgi-bin/query.pl?terms=hsa-miR-5688)  [hsa-miR-7-1-3p](http://www.mirbase.org/cgi-bin/query.pl?terms=hsa-miR-7-1-3p)  [hsa-miR-7-2-3p](http://www.mirbase.org/cgi-bin/query.pl?terms=hsa-miR-7-2-3p) |
|  |  | |  |
| [rs11053594](http://www.ncbi.nlm.nih.gov/SNP/snp_ref.cgi?rs=rs11053594) | C | | [hsa-miR-146b-3p](http://www.mirbase.org/cgi-bin/query.pl?terms=hsa-miR-146b-3p)  [hsa-miR-18a-3p](http://www.mirbase.org/cgi-bin/query.pl?terms=hsa-miR-18a-3p)  [hsa-miR-3173-5p](http://www.mirbase.org/cgi-bin/query.pl?terms=hsa-miR-3173-5p)  [hsa-miR-6799-3p](http://www.mirbase.org/cgi-bin/query.pl?terms=hsa-miR-6799-3p)  [hsa-miR-7976](http://www.mirbase.org/cgi-bin/query.pl?terms=hsa-miR-7976)  [hsa-miR-874-3p](http://www.mirbase.org/cgi-bin/query.pl?terms=hsa-miR-874-3p) |
|  | T | | [hsa-miR-10a-5p](http://www.mirbase.org/cgi-bin/query.pl?terms=hsa-miR-10a-5p)  [hsa-miR-10b-5p](http://www.mirbase.org/cgi-bin/query.pl?terms=hsa-miR-10b-5p) |
|  |  | |  |
| [rs11053595](http://www.ncbi.nlm.nih.gov/SNP/snp_ref.cgi?rs=rs11053595) | G | | [hsa-miR-146b-3p](http://www.mirbase.org/cgi-bin/query.pl?terms=hsa-miR-146b-3p)  [hsa-miR-18a-3p](http://www.mirbase.org/cgi-bin/query.pl?terms=hsa-miR-18a-3p)  [hsa-miR-3173-5p](http://www.mirbase.org/cgi-bin/query.pl?terms=hsa-miR-3173-5p)  [hsa-miR-6799-3p](http://www.mirbase.org/cgi-bin/query.pl?terms=hsa-miR-6799-3p)  [hsa-miR-7976](http://www.mirbase.org/cgi-bin/query.pl?terms=hsa-miR-7976)  [hsa-miR-874-3p](http://www.mirbase.org/cgi-bin/query.pl?terms=hsa-miR-874-3p) |
|  | T | | [hsa-miR-3661](http://www.mirbase.org/cgi-bin/query.pl?terms=hsa-miR-3661)  [hsa-miR-631](http://www.mirbase.org/cgi-bin/query.pl?terms=hsa-miR-631) |
|  |  | |  |
| [rs11053597](http://www.ncbi.nlm.nih.gov/SNP/snp_ref.cgi?rs=rs11053597) | C | | [hsa-miR-4715-5p](http://www.mirbase.org/cgi-bin/query.pl?terms=hsa-miR-4715-5p) |
|  |  | |  |
|  | |  |  |
