# Supplementary material for: A Comprehensive in Silico Analysis of Regulatory SNPs of Human CLEC7A Gene and Its Validation as Genotypic and Phenotypic Disease Marker in Recurrent Vulvovaginal Infections
Source: Front Cell Infect Microbiol. 2018 Mar 20;8:65. doi: 10.3389/fcimb.2018.00065 (PMC5869923; doi:10.3389/fcimb.2018.00065)
Supplement: Supplementary file 4 [file Table4.DOCX]

**Table S4** *CLEC7A* SNPs frequencies in present study population (North Indian) and modern human populations in human genomes variation projects including 1000 genome (https://www.ncbi.nlm.nih.gov/variation/tools/1000genomes/)

|  | Present study population | 1000 Genome Project | | | | | |
| --- | --- | --- | --- | --- | --- | --- | --- |
| SNP | North Indian  (N = 461) | ALL  (N = 2504) | AFR  (N = 661) | AMR  (N = 347) | EAS  (N = 504) | EUR  (N = 503) | SAS  (N = 489) |
| rs11053593 | | | | | | | |
| G | 0.82 | 0.82 | 0.64 | 0.93 | 0.87 | 0.93 | 0.86 |
| A | 0.18 | 0.17 | 0.36 | 0.07 | 0.12 | 0.07 | 0.13 |
| rs11053597 | | | | | | | |
| G | 0.81 | 0.82 | 0.64 | 0.93 | 0.87 | 0.93 | 0.86 |
| T | 0.19 | 0.17 | 0.36 | 0.07 | 0.12 | 0.07 | 0.13 |
| rs3901533 | | | | | | | |
| T | 0.57 | 0.53 | 0.50 | 0.42 | 0.81 | 0.24 | 0.67 |
| G | 0.43 | 0.46 | 0.49 | 0.58 | 0.18 | 0.75 | 0.32 |

ALL: all individuals; AFR: African; AMR: Ad Mixed American; EAS: East Asian; EUR: European; SAS: South Asian
